# NOseq: amplicon sequencing evaluation method for RNA m^6^A sites after chemical deamination

**DOI:** 10.1093/nar/gkaa1173

**Published:** 2020-12-11

**Authors:** Stephan Werner, Aurellia Galliot, Florian Pichot, Thomas Kemmer, Virginie Marchand, Maksim V Sednev, Tina Lence, Jean-Yves Roignant, Julian König, Claudia Höbartner, Yuri Motorin, Andreas Hildebrandt, Mark Helm

**Affiliations:** Institute of Pharmaceutical and Biomedical Sciences, Johannes Gutenberg-University Mainz, Staudingerweg 5, 55128 Mainz, Germany; Institute of Pharmaceutical and Biomedical Sciences, Johannes Gutenberg-University Mainz, Staudingerweg 5, 55128 Mainz, Germany; Institute of Pharmaceutical and Biomedical Sciences, Johannes Gutenberg-University Mainz, Staudingerweg 5, 55128 Mainz, Germany; Institute of Computer Science, Johannes Gutenberg-University Mainz, Staudingerweg 9, 55128 Mainz, Germany; Université de Lorraine, CNRS, INSERM, Epitranscriptomics and Sequencing (EpiRNA-Seq) Core Facility, UMS2008/US40 IBSLor, Biopôle UL, F-54000 Nancy, France; Institute of Organic Chemistry, Julius Maximilian University Würzburg, Am Hubland, 97074 Würzburg, Germany; Institute of Molecular Biology, Ackermannweg 4, 55128 Mainz, Germany; Institute of Pharmaceutical and Biomedical Sciences, Johannes Gutenberg-University Mainz, Staudingerweg 5, 55128 Mainz, Germany; Institute of Molecular Biology, Ackermannweg 4, 55128 Mainz, Germany; Génopode - Center for Integrative Genomics, Université de Lausanne, 1015 Lausanne, Switzerland; Institute of Molecular Biology, Ackermannweg 4, 55128 Mainz, Germany; Institute of Organic Chemistry, Julius Maximilian University Würzburg, Am Hubland, 97074 Würzburg, Germany; Université de Lorraine, CNRS, UMR7365 IMoPA, Biopôle UL, F-54000 Nancy, France; Institute of Computer Science, Johannes Gutenberg-University Mainz, Staudingerweg 9, 55128 Mainz, Germany; Institute of Pharmaceutical and Biomedical Sciences, Johannes Gutenberg-University Mainz, Staudingerweg 5, 55128 Mainz, Germany

## Abstract

Methods for the detection of m^6^A by RNA-Seq technologies are increasingly sought after. We here present NOseq, a method to detect m^6^A residues in defined amplicons by virtue of their resistance to chemical deamination, effected by nitrous acid. Partial deamination in NOseq affects all exocyclic amino groups present in nucleobases and thus also changes sequence information. The method uses a mapping algorithm specifically adapted to the sequence degeneration caused by deamination events. Thus, m^6^A sites with partial modification levels of ∼50% were detected in defined amplicons, and this threshold can be lowered to ∼10% by combination with m^6^A immunoprecipitation. NOseq faithfully detected known m^6^A sites in human rRNA, and the long non-coding RNA MALAT1, and positively validated several m^6^A candidate sites, drawn from miCLIP data with an m^6^A antibody, in the transcriptome of *Drosophila melanogaster*. Conceptually related to bisulfite sequencing, NOseq presents a novel amplicon-based sequencing approach for the validation of m^6^A sites in defined sequences.

## INTRODUCTION

**Figure 1.**
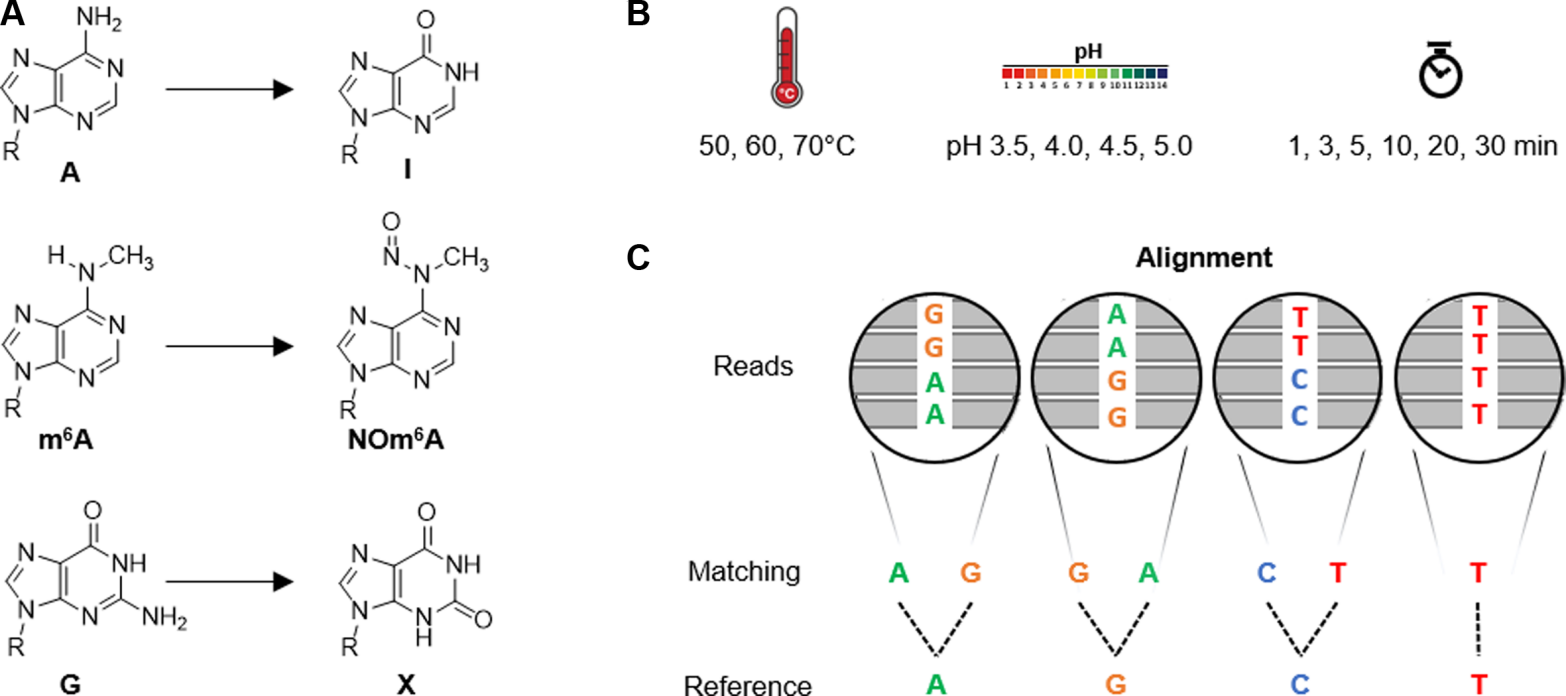
Deamination treatment and deamination sequencing (NOseq) output. (**A**) Reaction of adenosine, N^6^-methyladenosine (m^6^A) and guanosine under deamination treatment and their corresponding reaction products inosine, *N*^6^-methyl-*N*^6^-nitrosoadenosine (NOm^6^A) and xanthosine. (Supplement Figure S1 for cytidine and uridine). (**B**) Deamination treatment of a 53mer, containing m^6^A at position 33, with 72 different conditions (composed of different reaction temperatures, pH and reaction times) (Supplement Figure S4 for additional information). (**C**) Partial deamination sequencing output and required alignment adaptations (Example: native adenosine residues gave rise to either A or G signals, the latter deriving from the fraction having undergone deamination to I).

The occurrence of m^6^A in eukaryotic polyadenylated RNA is among the most investigated phenomena in recent RNA research. As we now know, m^6^A deposition on nascent RNA is an early and important event in maturation of mRNA, which is orchestrated by a sophisticated catalytic complex, whose components are subject to continuous updates (1–3). Methylation of adenosines has been shown to be important for regulation of downstream events including splicing (4), transport (5,6), degradation (7) and translation (8). Variegated amounts or lack of m^6^A have been associated with numerous pathologies (9–12), and impact in infection biology is a much-investigated aspect (13,14). The presence of m^6^A in mRNA has been known for decades (15–17), pioneered about 50 years ago by analytical techniques that are still widely used, namely isolation of mRNA by hybridization to poly(dT)-cellulose (18,19) or poly(U)-sepharose (20) and enrichment of m^6^A-containing RNA using antibodies (21,22). Breakthrough papers in 2012 (23,24) combined this approach with deep sequencing to create the first m^6^A maps of what is now termed the ‘epitranscriptome’ (25), thus creating a boost of the field that is still unabated (26,27). However, the community is in agreement, that the use of antibodies alone, or in refining combinations with other techniques (15–17), still does not provide quantitative data at single nucleotide resolution (28). Numerous tools from chemical biology have been applied to the task, including catalytic DNA (29), chemically altered dNTPs (30), engineered SAM analogues (31) or engineered polymerases (32) for the generation of a reverse transcription signature, or derivatives of SAM or metabolic precursor methionine (33) for click chemistry-based enrichment. Current developments focus on the use of nanopore technology (34), m^6^A-discriminating nucleases (35–37) and fusion proteins for RNA-editing adjacent to m^6^A sites (38,39). Despite these developments, a technology that resembles the bisulfite sequencing of m^5^C in terms of resolution and quantification is not yet in sight. Taking a clue from the chemistry of bisulfite sequencing (40–42), we examined the possibility of a reagent that would deaminate adenosines to inosine (I), and thus change its sequencing properties, while differentially reacting with m^6^A such as to not alter its reverse transcription signal. While adenosines are largely inert to nucleophiles like bisulfite, we found reports on adenosine deamination by nitrous acid (43), which led to the characterization of *N*^6^-methyl-*N*^6^-nitrosoadenosine (NOm^6^A) (44) (Figure 1A), with unknown base-pairing properties. For a targeted site-specific investigation of modified nucleotides in biological templates, a common methodology is the amplification of sequence regions. By applying primers to define sequence contexts, amplicon libraries for deep sequencing can be prepared and subsequently analysed (45).

Here, we present NOseq, an Illumina sequencing-based analysis pipeline for deaminated RNA sequences and m^6^A site detection by amplicon sequencing. The workflow combines an optimized deamination protocol, target-specific library preparation and a deamination-related alignment algorithm. The latter is based on the well-known seed-and-extend mapping strategy, first introduced in Blast (46) and mostly used by short read analysis tools (47) as SOAP (48) and BOWTIE (49). The algorithm principle stands on the extension and selection of matching *k*-mers (short sequence of *k* bases) between reads and references. Basically, an alignment score is attributed according to a substitution matrix, taking mismatches and gaps into account. Here, we implemented an asymmetric substitution matrix considering deamination-induced nucleotide conversion, an alignment strategy known from bisulfite sequencing (e.g. BSMAP (50)). We successfully recapitulated known m^6^A sites in human MALAT1 lncRNA, human 18S rRNA and validated new m^6^A sites in mRNA *Hairless* (H) and *female-lethal-2-d* (fl(2)d) from *Drosophila melanogaster*.

## MATERIALS AND METHODS

### RNA preparation

#### Total RNA extraction from HEK and HeLa cells

HEK293 or HeLa cells were cultured at 37°C with 5% CO_2_ in T-160 cell culture flasks containing growth medium (90% D-MEM, 10% FBS and 1% Pen/Strep). Cultures at around 80% confluency were routinely splitted, i.e. every 24–48 h in new growth medium until harvesting of the cells and up to 1 month (10 passages). For total RNA extraction, cells were detached from the flasks for 5 min with 3 ml of Trypsin/EDTA (Sigma-Aldrich) and pelleted by low speed centrifugation in a 50 ml tube. The cells at confluency were evaluated (seeding) at ∼18 million, resuspended and vortexed in 2 ml TriReagent (Sigma Aldrich) before adding 800 μl of chloroform allowing phase separation under centrifugation (12 000 × g, 4°C, 15 min). The upper aqueous phase containing RNA was precipitated with 250 μl of 100% nuclease-free isopropanol. After vortexing, incubation and centrifugation (15 000 × g, 4°C, 10 min), the pellet was washed twice with 80% ethanol, centrifuged again and the air-dried pellet was finally dissolved in MilliQ water.

#### PolyA RNA extraction from Drosophila melanogaster

*Drosophila melanogaster* Oregon-R wild-type strain and *Ime4^null^* knockout strain (51) were maintained at 25°C, at standard conditions. For total RNA isolation, 20 flies were smashed together per 1.5 ml tube with 100 μl TriReagent (Sigma Aldrich) before adding another 400 μl and incubating the mixture at 4°C for 5 min. After adding 100 μl chloroform and vortexing for 15 s, an incubation step at 4°C for 10 min was followed by centrifugation (12 000 × g, 4°C, 15 min), permitting phase separation. The upper aqueous phase was kept for total RNA precipitation by adding 250 μl of 100% nuclease-free isopropanol. After centrifugation (15 000 × g, 4°C, 10 min), the RNA pellet was washed twice with 750 μl 80% ethanol and centrifuged again. Ethanol was then removed, and the pellet air-dried for 5 min at room temperature. The pellet was finally dissolved in MilliQ water and quantified. For polyA RNA extraction, 60 μg of total RNA were first treated with DNase I (Thermo Scientific) to avoid DNA contamination before incubation with 100 μl washed oligo d(T)25 magnetic beads (New England Biolabs), to isolate mRNA from total RNA according to a modified protocol of Dynabeads (Thermo Scientific), including a second round of purification and elution in MilliQ water.

#### RNA quantification and quality control

RNA was quantified using the UV-Vis spectrophotometer Nanodrop 2000 (Thermo Scientific) and the purity was assessed according to both absorption ratios *A*_260_/*A*_280_ and *A*_260_/*A*_230_ respectively in the range of 2 and 1.8–2.2 reflecting contaminant free samples. Isolated total RNA and mRNA were analysed, and quality evaluation by automated electrophoresis on the Agilent 4200 TapeStation system, following RNA ScreenTape Assay instructions, was performed.

#### Methylated RNA immunoprecipitation (MeRIP)

The anti-m^6^A monoclonal mouse antibody (purified IgG) (Synaptic Systems) was first incubated overnight at 4°C in 1× PBS with Protein G Sepharose 4 Fast Flow antibody purification resin (GE Healthcare). Around 8 μg of total RNA, mRNA or synthetic oligonucleotide (Table 1) were added to the binding buffer after several antibody-beads washing steps and kept for 2 h incubation at 4°C and under shaking. This incubation step was repeated, including the washing steps, together with the addition of 0.5 mg/ml adenosine (Sigma-Aldrich) in order to induce a competitive elution of non-targeted RNA. Targeted m^6^A RNA fragments were then isolated from the antibodies by TriReagent (Sigma Aldrich) and following ethanol precipitation.

**Table 1.** RNA oligonucleotides (purchased from IBA Lifesciences, Germany)

Name	Sequence
53mer (m^6^A_33_)	5′-AUAGGGGAAUGGGCCGUUCAUCUGCUAAAAGG(m^6^A)CUGCUUUUGGGGCUUGUAGU-3′
53mer (A_33_)	5′-AUAGGGGAAUGGGCCGUUCAUCUGCUAAAAGG(A)CUGCUUUUGGGGCUUGUAGU-3′

### Deamination

#### Deamination with sodium nitrite

RNA (synthetic/isolated/immunoprecipitated) was deaminated under different conditions. Reactions were performed in a total volume of 100 μl, including RNA, 1 M sodium acetate/acetic acid buffer (pH 3.5, 4.0, 4.5 or 5.0) and 1 M sodium nitrite solution (final concentrations) at 50, 60 or 70°C for 1, 3, 5, 10, 20 or 30 min. The reaction was stopped by adding 0.5 M ammonium acetate solution (final concentration) and 1 μl glycogen (Thermo Scientific) to a final volume of 250 μl. By adding 750 μl ethanol (−80°C) the treated RNA was ethanol precipitated for 1 h at –80°C. Samples were centrifuged for 45 min at 5° and 15 000 × g. Pellets were washed with 80% ethanol and centrifuged again. Then, the ethanol precipitation step was repeated. Pellets were resuspended with 10 μl MilliQ water.

#### Deamination with diethylene glycol dinitrite

Diethylene glycol dinitrite was synthesized in analogy to a known procedure (52) as follows. A mixture of diethylene glycol (54 g; 0.5 mol), sodium nitrite (69 g; 1.0 mol) and ice (100 g) was placed into a one-liter glass beaker. Conc. hydrochloric acid (101 g; 0.5 mol) was added dropwise under vigorous stirring and cooling with an ice bath so that the reaction temperature did not exceed 5°C. Afterwards, the reaction mixture was stirred for 5 min and then transferred to a separating funnel. The organic layer was separated, washed with diluted aq. potassium carbonate and dried with potassium carbonate to give 34.4 g (42%) of the title product as a yellow oil. 1H NMR (400 MHz, CDCl_3_): δ = 3.77 (m, 4H, 2 × CH_2_), 4.86 (m, 4H, 2 × CH_2_) ppm. 13C NMR (125 MHz, CDCl_3_): δ = 67.2, 69.3 ppm.

Synthetic RNA was deaminated by incubation in an aqueous solution containing 1 M diethylene glycol dinitrite, 2 M pyridine and 1 M potassium thiocyanate (final concentrations) for 2, 5 or 10 h at 37°C. The solution was diluted with buffer (10 mM Tris pH 7.4, 300 mM sodium chloride, 1 mM ethylenediaminetetraacetic acid) and the RNA was precipitated by addition of three volumes of cold ethanol and incubation in liquid nitrogen for 15 min. After centrifugation and washing with 75% ethanol, the pellet was resuspended in MilliQ water, and the integrity of the RNA was checked by analytical PAGE (stained by SYBR gold).

### LC-MS

LC-MS/MS analysis was used to monitor the deamination levels of treated RNA samples. Prior to LC-MS/MS analysis, RNA samples (were digested into nucleosides according to the following protocol: samples were incubated in a buffer of 25 mM ammonium acetate (pH 7.5), 0.3 U nuclease P1, 0.1 U snake venom phosphodiesterase, 1 U fast alkaline phosphatase and 10 U Benzonase overnight at 37°C. The digested samples were analysed on an Agilent 1100 HPLC series equipped with a diode array detector (DAD) and an Agilent 1100 mass selective detector (LC/MSD-Trap). A YMC Triart C18 column (S-3 μm/12 nm, 120 Å, column size: 150 × 3.0 mm I.D.) from YMC Europe (Germany) was used at 30°C column temperature. Mobile phase A (MPA) consisted of 5 mM ammonium acetate buffer adjusted to pH 5.4 using acetic acid and mobile phase B (MPB) of pure acetonitrile. The elution was performed at a flow rate of 0.4 ml/min using the following gradient: 3% MBP from 0 to 3 min, 3–15% MPB from 3 to 30 min, 15–90% MPB from 30 to 31 min, 90% MBP from 31 to 35 min, 90–3% MPB from 35 to 36 min and 3% MPB from 36 to 45 min. The effluent from the column was measured photometrically at 254 nm by the DAD for the detection of the nucleosides. In addition to the UV detection, a mass spectrometer was used for the identification of nucleosides, e.g. *N*^6^-methyl-*N*^6^-nitrosoadenosine (NOm^6^A). Mass spectrometer settings used included ion polarity: positive; ion Source: ESI; dry temp: 350°C; nebulizer: 15 psi; dry gas: 12 l/min; trap drive: 43.0; Octapole RF amplitude: 140.3 V_pp_; capillary exit: 96.6 V; skimmer: 40.0 V; Oct 1 DC: 12.0 V; Oct 2 DC: 1.7 V; scan range: 105–600 *m*/*z*; averages: two spectra; maximum accumulation time: 200 ms; and ICC target: 30 000. Data was analysed with Bruker LC-MS data reading software (DataAnalysis) and nucleosides were monitored by multiple reaction monitoring (dynamic MRM mode).

### Library preparation and sequencing

#### Synthetic oligonucleotide

Around 500 ng of (treated) synthetic RNA oligonucleotide (Table 1) were used as starting material for library preparation. This includes all synthetic oligonucleotide samples, used directly as delivered, mixed or immunoprecipitated before library preparation. First, a two-step end-repair was performed, combining 5′- and 3′-dephosphorylation (Antarctic Phosphatase) and subsequent 5′-phosphorylation (Polynucleotide Kinase PNK), according to the manufacturer's instructions (New England Biolabs) and with addition of an RNase inhibitor (RNaseOUT, Invitrogen). The end-repaired RNA was purified with RNeasy MinElute Cleanup kit (Qiagen), using a larger quantity of ethanol to recover smaller RNA fragments. Library preparation was then prepared following the NEBNext Small RNA Library kit for Illumina (New England Biolabs): after ligation of the first adapter at the 3′-end of the end-repaired RNA, the reverse transcription primer was hybridized, followed by the second adapter ligation at the 5′-end of the RNA. The reverse transcription (RT) was performed, using ProtoScript II (in general) or SuperScript IV (for RT test—Supplement Figure S11), during 1 h at 50°C, according to the manufacturer's manual, directly followed by polymerase chain reaction (PCR) employing LongAmp Taq polymerase during 15 cycles at a primer annealing temperature of 62°C. The indexing of the samples takes place at this step: a P7 primer was chosen among the 48 differently barcoded primers from the NEBNext Multiplex Small RNA Library Prep Set for Illumina (New England Biolabs). The P7 and P5 primer allow amplification of the templates and implementation of the necessary sequences for Illumina sequencing. A final clean-up and size selection step were performed to purify the library, applying the GeneJet PCR kit, according to the supplier's instructions.

#### Amplicon sequencing with biological samples

Around 500 ng of (treated) RNA (messenger or total RNA) were incubated at 75°C for 5 min with 2.5 μM of targeted RT primers (Table 2). Reverse transcription was performed, using 10 U ProtoScript II (New England Biolabs) in addition with 0.5 mM dNTP mix, 10 mM DTT, 0.4 U RNase inhibitor, 1x ProtoScript II Buffer (final concentrations) and MilliQ water for 2 h at 50°C. Then, polymerase chain reaction (PCR) was performed by mixing 5 U of LongAmp Taq DNA Polymerase (New England Biolabs) with 0.4 μM of targeted P5 primer (Table 2) and 0.4 μM P7 primer from the NEBNext Multiplex Small RNA Library Prep Set for Illumina (New England Biolabs) in addition to 300 mM dNTP mix, 1x LongAmp Taq Reaction Buffer (final concentrations) and MilliQ water. PCR was performed for 25 cycles at a primer annealing temperature of 50°C. The amplicon obtained was analysed *via* 10% denaturing polyacrylamide gel electrophoresis for 40 min at 10 W. The corresponding gel area (amplicon size), determined by GeneRuler Low Range DNA Ladder (Thermo Scientific), was cut out and eluted. The gel elution was performed overnight at 25°C in 0.5 M ammonium acetate solution, followed by Nanosep filtering (0.45 μm, VWR) and subsequent ethanol precipitation.

**Table 2.** DNA oligonucleotides for amplicon sequencing (purchased from IBA Lifesciences, Germany and Biomers, Germany)

Name	Sequence
Malat1 RT primer	5′-AGACGTGTGCTCTTCCGATCTNNNNNNNTGCTAGTCCTCAGGA-3′
Malat1 P5 primer	5′-AATGATACGGCGACCACCGAGATCTACACACACTCTTTCCCTACACGACGCTCTTCCGATCTAAGATCAAGAGTAAT-3′
18S RT primer	5′-AGACGTGTGCTCTTCCGATCTNNNNNNNCCTTCCGCAGGTTCA-3′
18S P5 primer	5′-AATGATACGGCGACCACCGAGATCTACACACACTCTTTCCCTACACGACGCTCTTCCGATCTGACGGTCGAACTTGA-3′
H RT primer	5′-AGACGTGTGCTCTTCCGATCTNNNNNNNCATATTCTTATTGCA-3′
H P5 primer	5′-AATGATACGGCGACCACCGAGATCTACACACACTCTTTCCCTACACGACGCTCTTCCGATCTCGGCCCGCCGTGTGT-3′
fl(2)d RT primer	5′-AGACGTGTGCTCTTCCGATCTNNNNNNNGTTTTGCTCGTATTT-3′
fl(2)d P5 primer	5′-AATGATACGGCGACCACCGAGATCTACACACACTCTTTCCCTACACGACGCTCTTCCGATCTGTCGCTGCAATGACT-3′

#### DNA library quantification and quality control

DNA libraries were quantified using the Qubit dsDNA HS (High Sensitivity) Assay Kit with the Invitrogen benchtop fluorometer Qubit 2.0. Each library (amplicon and synthetic oligonucleotide) was analysed, and quality evaluation by automated electrophoresis on the Agilent 2100 Bioanalyzer system was performed, following the Bioanalyzer High Sensitivity DNA Kit instructions.

#### Sequencing method

Libraries were subjected to high-throughput sequencing using an Illumina MiSeq instrument with single-read (SR50) or paired-end (PE75) mode. Prior to loading on the sequencing chip, the multiplexed libraries were diluted to 6–8 pM final concentration.

#### miCLIP

miCLIP was performed as described previously (16) using 10 μg of purified mRNA from *Drosophila melanogaster* S2R+ cells and 5 μg of anti-m^6^A antibody (Synaptic Systems). Immunoprecipitations were performed in quadruplicates and as a control one immunoprecipitation was performed where UV-crosslinking was omitted. Of note, this sample produced a library of limited complexity, reflecting a low amount of background mRNA binding. Briefly, total RNA was isolated using Trizol reagent (Invitrogen) and DNA was removed with DNase-I treatment (NEB). Polyadenylated RNA was purified by two rounds of binding to Oligo (dT)25 magnetic beads (NEB) and fragmented with RNA fragmentation solution (Ambion) using 1 μl of solution per 2 μg of mRNA and with 7 min incubation at 70°C. Immunoprecipitation was performed at 4°C in 500 μl of binding buffer (BB) (50 mM Tris–HCl pH 7,4, 150 mM sodium chloride, 0,5% NP-40). First, isolated mRNA and antibody were incubated for 2 h. Samples were then transferred to individual well of a 12-well cell culture plate and crosslinked on ice (two-times at 150 mJ/cm^2^). Next, 60 μl of magnetic ProteinG beads (Invitrogen) were resuspended in 500 μl of BB and added to the IP sample. Samples were then incubated for additional 2 h at 4°C, before washing with ice-cold solutions was performed: 1× with BB, 2× with high salt buffer (50 mM Tris–HCl pH 7,4, 1 M sodium chloride, 1% NP-40, 0,1% sodium dodecyl sulfate), 1× BB, 2× with PNK buffer (20 mM Tris–HCl pH 7,4, 10 mM magnesium chloride, 0,2% Tween). All washes were performed by gentle pipetting and with 1 min incubation on ice. Washes with HSB were additionally rotated for 2 min at 4°C. Finally, beads were resuspended in 900 μl of PNK buffer. 40 μl were used for WB analysis to evaluate immunoprecipitation efficiency. Remaining 860 ml were used for library preparation. All steps of library preparation were performed as previously described in Sutandy *et al.* (53). Libraries were amplified with 17 PCR cycles and sequenced on a NextSeq500 with a read length of 120 bp, single end (Table 3).

**Table 3.** DNA oligonucleotides for miCLIP (purchased from Integrated DNA Technologies, Inc., USA)

Name	Sequence
L3-Linker	5′-rApp-AGATCGGAAGAGCGGTTCAG-ddC-3′
miCLIP_IP1	5′-P-NNCTCGNNNAGATCGGAAGAGCGTCGTGGATCCTGAACCGC-3′
miCLIP_IP2	5′-P-NNTGTGNNNAGATCGGAAGAGCGTCGTGGATCCTGAACCGC-3′
miCLIP_IP3	5′-P-NNTTTCNNNAGATCGGAAGAGCGTCGTGGATCCTGAACCGC-3′
miCLIP_IP4	5′-P-NNCGATNNNAGATCGGAAGAGCGTCGTGGATCCTGAACCGC-3′
miCLIP_ctrl	5′-P-NNGACCNNNAGATCGGAAGAGCGTCGTGGATCCTGAACCGC-3′

### Bioinformatic analysis

#### NOseq alignment

NOseq Illumina output demultiplexed FastQ files were unzipped. The nucleotide sequences (FASTA format), read information (identity number) and their corresponding quality scores (encoded in ASCII) were extracted, compiled and analysed using Python. The bioinformatic pipeline is single file dependent and each file is associated to a reference sequence. Prior to the alignment of the reads, the whole reference sequence was divided into smaller overlapping segments with identical size (*k*-mer of size *k* = 11 per default) and indexed by starting position. In order to increase the algorithm speed, *k*-mers were alphabetically ordered. The alignment process is relying on seed-and-extend strategy. Sequenced reads were individually segmented into *k*-mers of size *k*, scanned against the reference sequence *k*-mers and scored according to the asymmetric substitution matrix (Supplement Figure S5). In case of matching seeds, the alignment was then extended as well as the score associated. Regarding the +1/−1 reward/penalty system and by allowing mismatches according to partial deamination (Figure 1), only the reads with a score equal to their length were kept. Furthermore, in order to avoid shifting of the reading frame, sequencing errors and to have a homogeneous coverage, only reads starting at the first nucleotide of the reference sequence were selected for further evaluation. In addition, this strict selection process is applied in order to limit the occurrence of NGS errors within the short sequencing reads (54). Finally, the totality of perfectly aligned reads was used for alignment analysis.

#### NOseq data analysis

**Figure 2.**
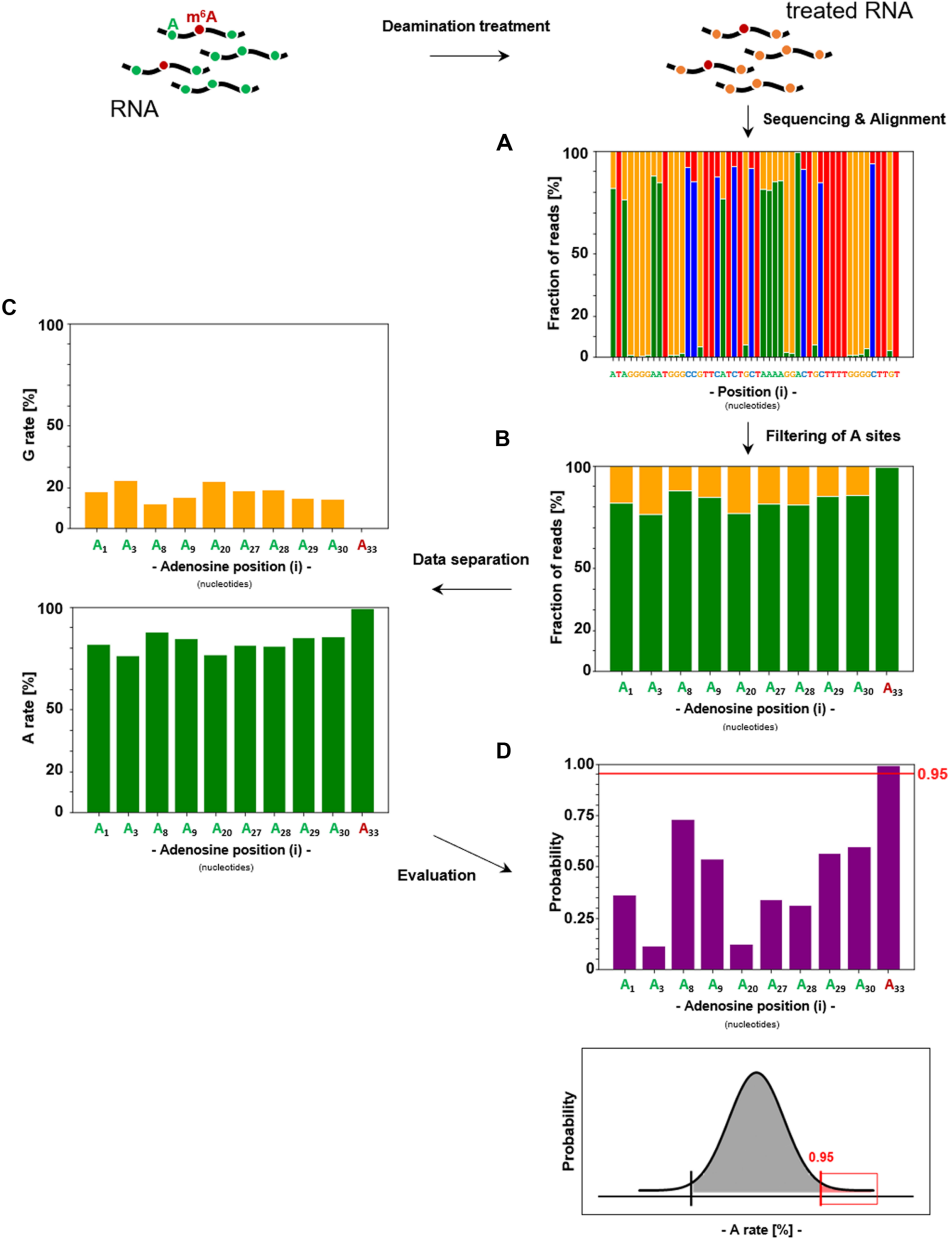
General analysis pipeline of NOseq data (exemplary for deamination of the 53mer with a treatment of 50°C, pH 4.0 and 20 min). After deamination treatment the treated RNA is used for library preparation and sequenced (for comparison to two-step alignment - see Supplement Figure S17). (**A**) Reads are aligned to the reference with an asymmetrical substitution matrix (see Supplement Figure S5) to address partial deamination and the corresponding fraction of reads in % is plotted for each position in an alignment plot (A in green, G in orange, C in blue and T in red). (**B**) Adenosine sites are filtered from the alignment plot. (**C**) The fraction of reads in % is separated to generate adenosine and guanosine rate plots (separated adenosine and guanosine fraction of reads in % at A sites). (**D**) The A rate is then used for evaluation of the m^6^A site in a probability plot, which illustrates the probability of an A site being an m^6^A, calculated by the distance of the respective A rate to the average A rate, with a red line at 0.95 showing the detection threshold.

From the aligned reads, the number of adenosine (A), cytidine (C), guanosine (G) and thymidine (T) was summed for each position and corresponding frequencies were plotted using matplotlib python library. In addition, only A positions in the reference sequence were selected and associated A and/or G rates were plotted. From this analysis, the cumulative density function of the normal distribution was calculated for each A position using the python package scipy.stats.norm and plotted together with the detection threshold at 0.95 (red line) (see Figure 2 as example). Above this limit, 5% of the most distant A rates to the overall distribution was observed, highlighting A sites with a high probability to behave differently to the other positions, i.e. presenting m^6^A candidate sites under deamination conditions.

#### NOseq UMI assessment

NOseq data were analysed in order to determine the read redundancy by assessment of the unique molecular identifiers (UMI). The UMI sequence (7 nt) of every aligned read was determined and the dataset was checked for duplicates (reads with same UMI sequences).

#### miCLIP data analysis

miCLIP Data analysis was performed as previously described in Linder *et al.* (16).

## RESULTS

### Screening of deamination conditions by LC-MS and sequencing

In order to distinguish m^6^A from unmethylated adenosines in sequencing data, the major step was the conversion of adenosines into inosines to change the base-pairing properties in reverse transcription. According to reports about adenosine deamination by nitrous acid (43), we set up a deamination protocol for RNA and performed a condition screening with a synthetic 53mer containing m^6^A at position 33 (Table 1). After optimizing a range of temperature, incubation time and pH values, we identified conditions leading to complete deamination of RNA (70°C, pH 3.5, 60 min), as analysed by RP18 chromatography. Deamination included all exocyclic, primary amines, thus converting adenosines to inosines, cytidines to uridines, and guanosines to xanthosines (X) (55) in the process (Figure 1A and Supplement Figure S1). Using LC–MS, we detected, in addition to the above, a peak with an *m*/*z* = 311, indicating partial formation of *N*^6^-methyl-*N*^6^-nitrosoadenosine (NOm^6^A) from m^6^A (44) to a maximum of 50–70% (Supplement Figure S2). The deaminated synthetic RNA template was then subjected to library preparation and first sequencing attempts. Thereby we expected a change in base-pairing properties for all deaminated and converted nucleotides, with unknown properties for NOm^6^A (Supplement Figure S3). In the process of optimizing cDNA synthesis by reverse transcription and library preparation, it turned out that RNA degradation under the acidic deamination conditions rendered subsequent Illumina sequencing impracticable. However, by screening a set of in total 72 deamination conditions, resulting from permutation of different reaction temperatures, incubation times and pH values (Table 1, Figure 1B), we identified a deamination treatment that allowed successful library preparation and sequencing of the synthetic RNA template (Supplement Figure S4). A range of conditions leading to partial deamination of RNA could be determined (with A-to-I conversion between 10% and 50%), within which the sequencing output was optimized in terms of aligned reads and m^6^A signal strength, i.e. the data were evaluated for the fraction of aligned reads and maximum m^6^A signal intensity. Importantly, partial deamination prevented the use of conventional alignment algorithms. As shown in Figure 1C (and Supplement Figure S3), native adenosine residues gave rise to either adenosine or guanosine signals, the latter deriving from the fraction having undergone deamination to inosine. Similarly, cytidines yielded cytidine or thymidine signals, respectively. At guanosine positions in the reference sequence, the analysis yielded a vast majority of guanosine signals, with a small remainder of adenosine. Of note, only native m^6^A produced unaltered adenosine signals. These observations and the associated need for deliberately ambiguous alignments due to partial deamination required a specific alignment algorithm, since conventional mapping tools did not offer this capability.

### Alignment and analysis of NOseq data

To enable effective mapping of reads, from what we termed NOseq, an adapted mapping algorithm using a seed-and-extend strategy based on local alignment (46) was developed. At the core of this algorithm was a substitution matrix composed of score values that reward or penalize alignment options. We adapted these values to account for the possibility of mapping guanosine and adenosine onto any purine in the reference genome, and of thymidine (or uridine, respectively) onto any pyrimidine, as depicted in Supplement Figure S5. We obtained an alignment plot, showing the fraction of respective nucleotides for each position mapped along the reference sequence (Figure 2A). The samples were analysed by the degree of A-to-I conversion, expressed as the fraction of decreased adenosine and increased guanosine signals at adenosine (and prospective m^6^A) sites (Figure 2B). In all but the weakest and strongest deamination conditions, the m^6^A_33_ showed the highest adenosine and lowest guanosine (A-to-I deamination) signals. For further analysis we separated the adenosine and guanosine fractions into two individual plots, showing the adenosine and the guanosine rate respectively (Figure 2C). Using only reads that cover the entire sequence under investigation, the distance of the A rate (at each position) from the mean deamination yield (mean A rate) was used to identify potential m^6^A candidates. Thereby a conventional probability plot was applied to highlight positions with a signal, significantly different from other adenosine sites (Figure 2D). The m^6^A signal strength was then used to identify optimal parameters. In general, the weakest deamination conditions were not sufficient to achieve a distinction between A and m^6^A, and the strongest conditions led to non-informative data output, similar to non-deamination of the templates, although LC-MS analysis clearly documented near-complete deamination (Supplement Figure S6). We hypothesized that the product of the slowest deamination reaction, namely the guanosine-to-xanthosine conversion, might interfere with the RT-based amplification step, thus selecting exclusively those few RNA molecules that have escaped deamination. This hypothesis was confirmed by primer extension experiments on a synthetic RNA fragment, which showed, that xanthosine residues indeed significantly slow down and partially block reverse transcription (Supplement Figure S7). Given that each RNA contains an individual number of guanosine residues, we considered the possibility that optimal deamination strength might vary among different sequences under investigation. However, our follow-up experiments for target-specific evaluation of m^6^A sites by amplicon sequencing to investigate biological templates (*vide infra*), revealed that m^6^A sites can be detected in different sequence contexts with the same deamination treatment and analysis pipeline, without additional optimization steps. A probable explanation is that under the milder conditions used, the xanthosine blockage plays a minor role, since the guanosine conversion is slower in comparison to those of cytidine and adenosine (Supplement Figure S6). Therefore, the probability for multiple guanosine-to-xanthosine deamination events are indeed very low, even in G-rich stretches, and such species do not significantly contribute to the overall sequencing output. This assumption was also supported by our data on the synthetic oligo with multiple G-rich stretches (Supplement Table S1).

### m^6^A detection and threshold determination

**Figure 3.**
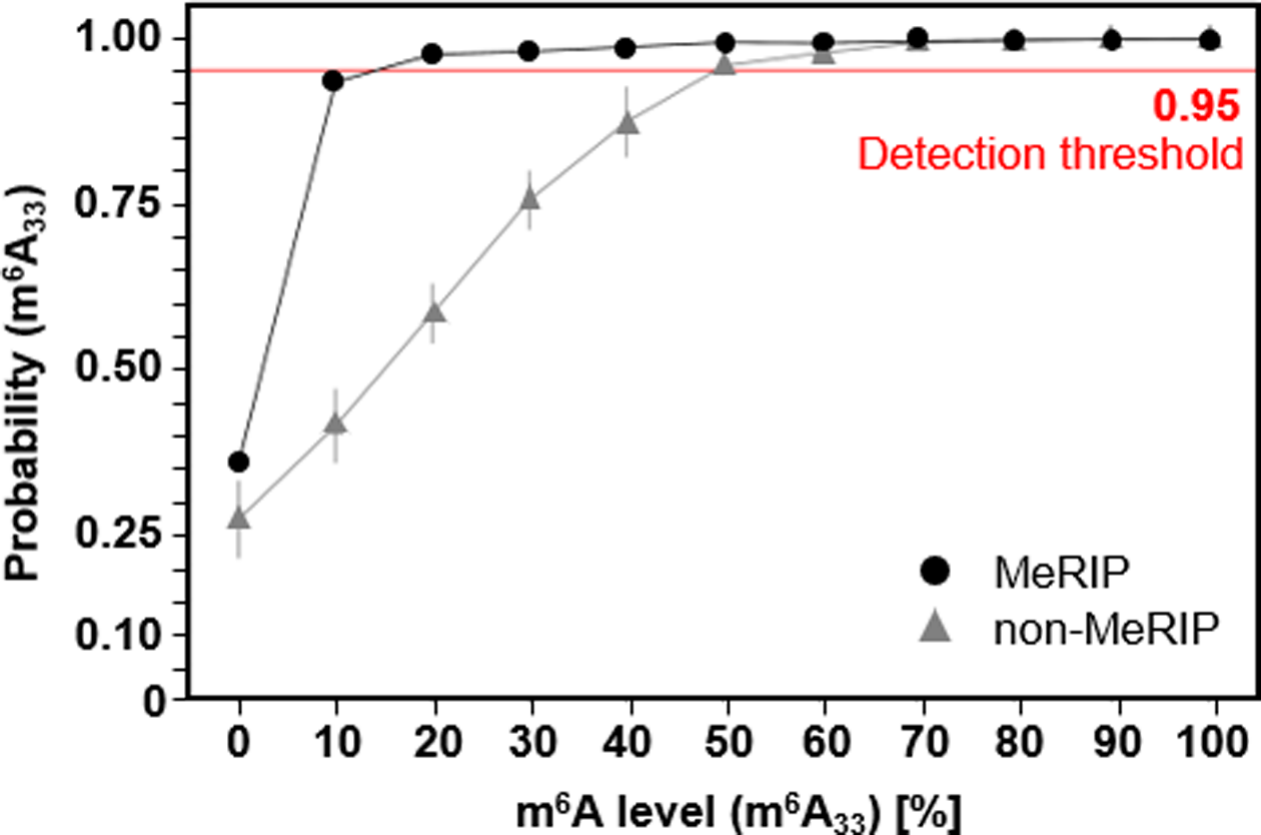
NOseq data comparison (MeRIP enriched vs. non-MeRIP) to address its detection threshold for sub-stoichiometric m^6^A with an m^6^A content at position 33 (m^6^A/A) of the 53mer that was varied from 0 to 100%. (black dots MeRIP, grey triangles non-MeRIP). Data was averaged from duplicates (error bars show standard deviations). Red line shows detection threshold at 0.95.

To evaluate the sensitivity of our method and address its detection threshold for sub-stoichiometric m^6^A levels at biological m^6^A sites, we performed a calibration by analysing the m^6^A signal in the same synthetic 53mer, now with an m^6^A content at position 33 (m^6^A/A) that was varied from 0 to 100%. Partial deamination parameters (50°C, pH 4.0, 20 min), representing a compromise featuring optimized mapping yield versus adenosine deamination, were chosen for this test (Figure 2). Furthermore, decreased formation of NOm^6^A (not shown) under these milder conditions is thought to avoid potential RT-arrest at m^6^A sites. NOseq data showed a statistically valid m^6^A signal in samples containing down to ∼50% m^6^A. This *de novo* detection limit could be lowered to ∼10% by conducting a methylated RNA immunoprecipitation (MeRIP) enrichment (56) prior to NOseq (Figure 3). Such detection on the basis of antibody-enriched RNA fragments was tested with regard to application of NOseq on low-abundant RNA species as mRNA. Expectedly, the linear dependence of m^6^A signal to m^6^A content is lost as a consequence of this enrichment step. The m^6^A signal in MeRIP-enriched RNA sequences therefore does not allow any conclusions to be drawn about the methylation level. However, once detected, a quantification of the m^6^A site in a subsequent step by using a series of mixtures of synthetic oligos with defined amounts of m^6^A seems practicable. Of such a calibration series, we determined the A signal at the m^6^A site in the deaminated calibration samples and put it into relation to the respective m^6^A content. Given the clearly linear correlation (Supplement Figure S8), we conclude that quantification of m^6^A sites might, in principle, be possible in a two-step procedure, where the first step must identify the position in a detection step, and the second step would require sequence-specific calibration for quantitative analysis. However, in contrast to straightforward validation of m^6^A in amplicons (*vide infra*), such a calibration would have to rely on synthetic modified RNA, and require considerable effort in terms of time and resources.

### Investigation and validation of biological m^6^A sites by amplicon sequencing

**Figure 4.**
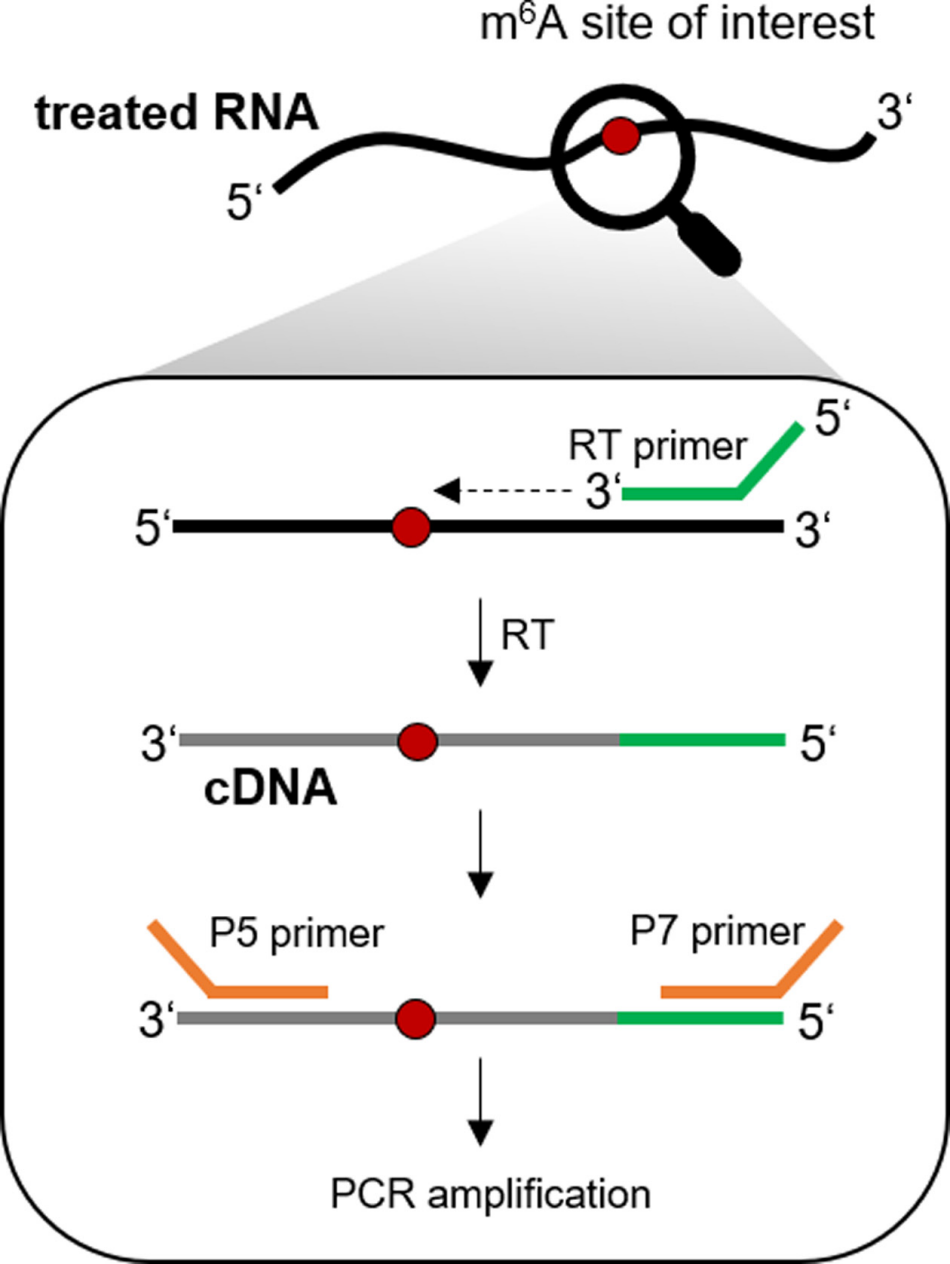
Library preparation scheme for amplicon sequencing of targeted m^6^A sites. The target region is amplified in a two-step process. First, reverse transcription of the deaminated RNA is performed with a target-specific RT primer. The latter contains the UMI and the binding sequence for the universal P7 primer including the barcode for Illumina sequencing. Afterwards, a target specific P5 primer and the P7 primer are used for PCR amplification.

We applied NOseq to five biological m^6^A sites to demonstrate the applicability of the method. To this end, we developed an amplicon sequencing method for target-specific evaluation of m^6^A sites (Figure 4). Of note, library preparation for the above calibration with synthetic RNA was based on an adapter ligation, while biological samples required the design of targeted primers, compatible with the Illumina sequencing technology, for each m^6^A site under investigation. In a two-step process, including reverse transcription and PCR amplification, sequencing libraries were prepared. To address partially deaminated RNA templates, we also tested primers with complementary degenerated target sequences. The extended binding possibilities yielded unspecific sequence amplification (not shown) and this strategy was therefore not further pursued. Rather, as a compromise of signal-to-noise *versus* sequencing output, we turned to conventional primers and used milder deamination conditions (60°C, pH 5.0, 10 min, Supplement Figure S9), thus obtaining sufficient PCR product for RNA-Seq. Assessment of unique molecular identifiers (UMIs) in typical amplicons (Supplement Figure S13 and Supplement Table S1) revealed an average read redundancy around 10% (Supplement Table S2), showing the samples to be representative and essentially free of significant PCR-amplification bias.

**Figure 5.**
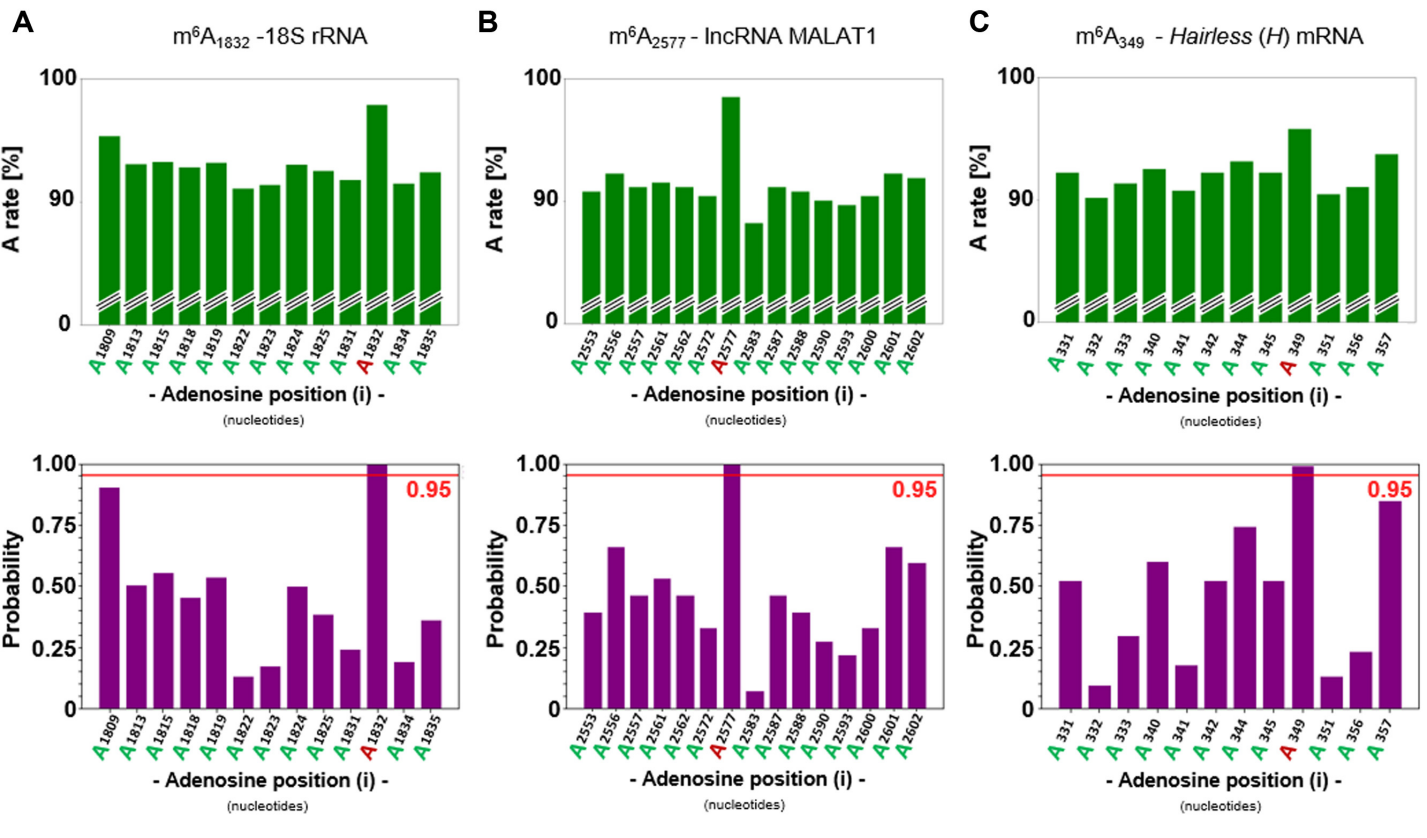
Adenosine rate (A fraction of reads in % at A sites) and probability plots of three biological amplicons, showing the progressed A-to-I conversion and corresponding m^6^A site evaluation (red line shows detection threshold at 0.95). (**A**) m^6^A at position 1832 in human 18S rRNA (HEK293T cells). (**B**) m^6^A at position 2577 in human lncRNA MALAT1 (HeLa cells). (**C**) m^6^A at position 349 in *Drosophila melanogaster* H mRNA after MeRIP enrichment. (Supplement Figures S14 & 15 for additional information, including non-treated references).

An initial experiment was targeted at the highly abundant and well-described m^6^A site at position 1832 in human 18S rRNA (57). Under the selected conditions we were able to clearly distinguish and detect the m^6^A site from the surrounding A sites (Figure 5A). Of note, plotting C-to-U (T-rate) and A-to-I (G-rate) changes across the amplicon sequence revealed a uniform distribution of deamination events, including the direct proximity to the m^6^A target (Supplement Figure S15). This demonstrated that the use of conventional primers did not incur deamination biases in the amplicon.

Without changes, these conditions for target-specific evaluation of m^6^A sites were subsequently applied to other m^6^A positions in different sequence contexts. As another m^6^A site for evaluation, the m^6^A at position 2577 in the human lncRNA MALAT1 was analysed. Again, the same conditions led to clear distinction between m^6^A and the surrounding A sites (Figure 5B). From the clear signals without prior MeRIP enrichment, the occupancy of the sites in rRNA and lncRNA was gauged to exceed 50% (determined detection threshold – Figure 3), which is in keeping with values obtained by other methods (57). As third validation, we applied NOseq in combination with MeRIP (56) to scrutinize m^6^A candidate sites in mRNA from *D. melanogaster*. The first investigated m^6^A candidate site was selected at position 349 in the 5′-untranslated region (UTR) of the protein-coding mRNA *Hairless* (*H*) in *D. melanogaster*, which plays a role in Notch signalling (58). This candidate site produced a strong signal in miCLIP experiments (Supplement Figure S10a) and was thus validated by NOseq as genuine m^6^A site (Figure 5c). Finally, a further validation was conducted on a sequence region comprising two m^6^A candidate sites drawn from miCLIP experiments (Supplement Figure S10b) in the coding sequence of the mRNA *female-lethal-2-d* (*fl(2)d*) in *D. melanogaster*, an associated component of the methyltransferase complex mediating m^6^A methylation (59). Both sites were detected *via* NOseq. As an ultimate validation, NOseq signals from these sites were compared to those obtained with RNA from an *Ime4* knockout mutant. We had previously characterized this knockout of the homolog of mammalian METTL3 protein, and found mRNA from this mutant to contain drastically reduced levels of m^6^A in polyA-RNA (51,60). Correspondingly, neither of the two m^6^A candidate sites were detectable by NOseq anymore in the respective amplicon (Supplement Figure S16), validating the detection of these m^6^A sites in mRNA.

## DISCUSSION

In summary, we developed an m^6^A detection approach based on chemical deamination of RNA. The pre-treatment with sodium nitrite in an acidic milieu and under heat influence enables discrimination of adenosines and m^6^A in RNA-Seq data. Chemical deamination of adenosines leads to a conversion to inosines and therefore to a certain number of guanosine signals in sequencing due to changes in reverse transcription properties. However, treatment does not lead to altered sequence information at m^6^A sites, which can hence be identified by lack of deamination products, corresponding to a high A rate, at a given position. Because xanthosine as a deamination product of guanosine effectively blocks reverse transcription, we had to resort to partial instead of complete deamination, as is known from bisulfite sequencing. Therefore, the method is not yet suitable for transcriptome-wide studies, but might be thus developed, if the xanthosine block can be overcome. One option could be a polymerase variant that bypasses xanthosine with higher efficiency. However, given that we already tested other RT enzymes, specific engineering or *in vitro* evolution might be necessary (32). Our method allows exchange of the RT enzyme without further adaptations (Supplement Figure S11). In this perspective, we have also successfully applied an organic nitrite as less acidic nitrosylation reagent (diethylene glycol dinitrite), with results similar to nitrous acid, but with decreased degradation (Supplement Figure S12). The detection of m^6^A sites with different methylation levels is possible, and the detection limit of the method can be further reduced by enrichment of m^6^A fragments with m^6^A-specific antibodies prior to the treatment, whereby even adenosines with very low methylation level (about 10%) can be differentiated.

A quantification of methylation stoichiometry in m^6^A sites subsequent to their validation by NOseq is made plausible by our proof-of-principle data on synthetic RNA. This would comprise synthetic template mixtures with ascending m^6^A content in the sequence context in question. The sequence-specific calibration differentiates the procedure from known methods (17). While plausible, it would admittedly require major efforts and an individual calibration for every m^6^A site in its particular sequence context. However, if corresponding calibration repetitions in different sequence contexts were producing consistently linear correlations, that might plausibly allow a transfer and application of linear calibrations (such as shown in Supplement Figure S8) from *bona fide* sites to new sites in question, and thus eliminate the need for subsequent sequence-tailored validations. However, the number of such analyses required for sufficient confidence is well outside the scope of this work.

Importantly, we here prove the applicability of our amplicon-based detection in as many as five biological m^6^A sites, two of them previously known, namely in human 18S rRNA and lncRNA MALAT1. Importantly, the other three led to validations of new sites in mRNA from *Drosophila melanogaster*. We anticipate that this approach will find application in the RNA research field by allowing site-specific m^6^A evaluation in various organisms and RNA species. In our experience, the method is easy to apply, and the specific primer target sequences can be easily adapted for other m^6^A sites under investigation (Supplement Figure S13). The required sequencing depth is rather low, with verified m^6^A detectability in our amplicon experiments based on a few hundred reads. Given a designed amplicon, an assay with modest requirements for experimental skills is readily established and enables straightforward comparative assessment of RNA samples in large numbers.

## DATA AVAILABILITY

The bioinformatic script and test datasets used for this study are available in the Supplementary Data (zip file).

Testdata: Sample1.fastq – 53mer (m^6^A_33_) – 50°C, pH 4.0, 20 min., Sample2.fastq – 18S rRNA amplicon – 60°C, pH 5.0, 10 min.

## Supplementary Material

gkaa1173_Supplemental_Files
