# Supplementary material for: NOseq: amplicon sequencing evaluation method for RNA m6A sites after chemical deamination
Source: Nucleic Acids Res. 2020 Dec 11;49(4):e23. doi: 10.1093/nar/gkaa1173 (PMC7913672; doi:10.1093/nar/gkaa1173)
Supplement: gkaa1173_Supplemental_Files [file gkaa1173_supplemental_files.zip › NOseq_Supplement_NAR_R2.pdf]

Supplement

List of abbreviations (selected)

lncRNA	long non-coding RNA
m^5C	5-methylcytosine
m^6A	*N*^{6}-methyladenosine
MeRIP	Methylated RNA Immunoprecipitation
miCLIP	m^6A Individual-nucleotide-resolution Cross-Linking and Immuno-Precipitation
mRNA	messenger RNA
NOm^6A	*N*^{6}-methyl-*N*^{6}-nitrosoadenosine
NOseq	m^6A deamination sequencing
PCR	Polymerase Chain Reaction
rRNA	ribosomal RNA
RT	Reverse Transcription
SAM	S-adenosylmethionine
SCARLET	Site-specific Cleavage And Radioactive-labelling followed by Ligation-assisted Extraction and Thin-layer chromatography

Tables

Table S1. *Investigated sequences and sequence regions (amplicon sequencing) for NOseq. m^{6}A candidate sites are shown in brackets.*

Name	Sequence
Oligo (m^{6}A$_{33}$)	5'-AUAGGGGAAUGGGCCGUUCAUCUGCUAAAAGG (m^{6}A) CUGCUUUUGGGGCUUGUAGU-3'
MALAT1 (m^{6}A$_{2577}$)	5'-UACCAA**C**UUAAUGUUUUUGCAUUGG (m^{6}A**)** CUUUGAGUUAAGAUUAUUUUUUAAA-3'
18S rRNA (m^{6}A$_{1832}$)	5'-CUAUCUAGAGGAAGUAAAAGUCGUA (m^{6}A) CAAGGUUUCCGTAGG-3'
H (m^{6}A$_{342}$)	5'-GCCUGUGAAAUUGGUUAAAUAACGU (m^{6}A) CAGUGGAAUGGGCCAAUGGCCAAAA-3'
fl(2)d (m^{6}A$_{451}$ & m^{6}A$_{472}$)	5'-AUGG (m^{6}A**)** CGAUCAAAGACCCUGUAUGA (m^{6}A**)** CAGUUACGACAAAAUGCCCCCGACC-3'

Table S2. *UMI analysis of NOseq amplicon sequencing data.*

Name	Read redundancy (%)
MALAT1 (m^{6}A$_{2577}$)	8 %
18S rRNA (m^{6}A$_{1832}$)	10 %
H (m^{6}A$_{342}$)	17 %
fl(2)d (m^{6}A$_{451}$ & m^{6}A$_{472}$)	5 %
Average	10 %

Figures

Figure S1

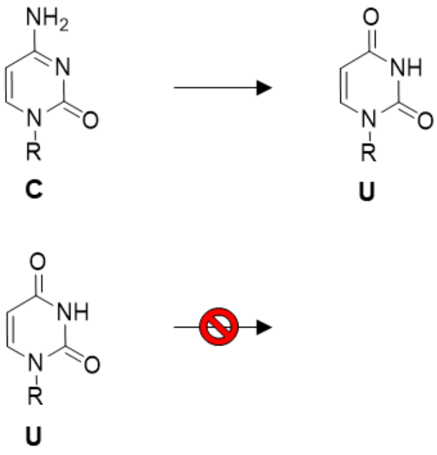

***Figure S1**. Reaction of cytidine and uridine under deamination treatment and their reaction products. (See Figure 1 for adenosine, m^{6}A and guanosine).*

Figure S2

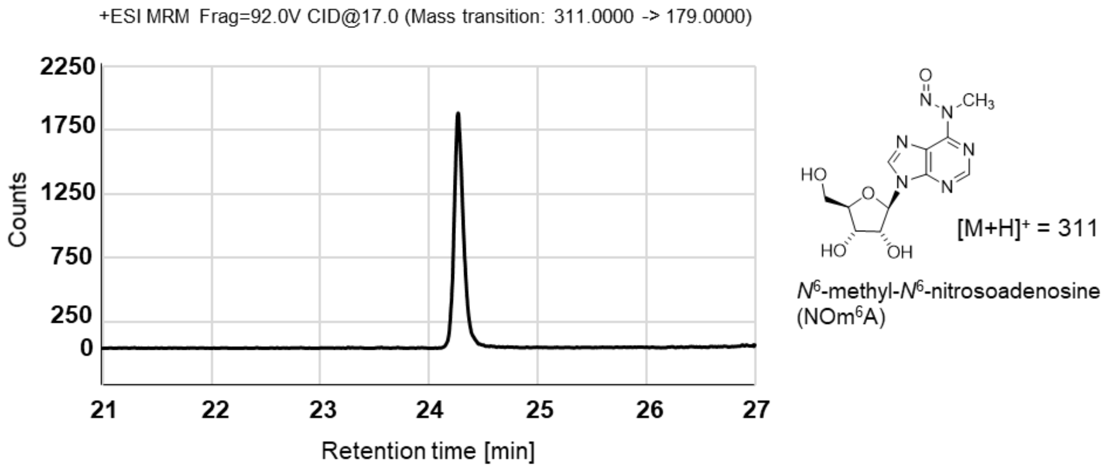

Figure S2. *Extracted ion chromatogram (EIC) from LC-MS analysis of N^6-methyl-N^6-nitrosoadenosine (NOm^{6}A), showing a peak at 24.3 min with a mass transition of 311 to 179.*

Figure S3

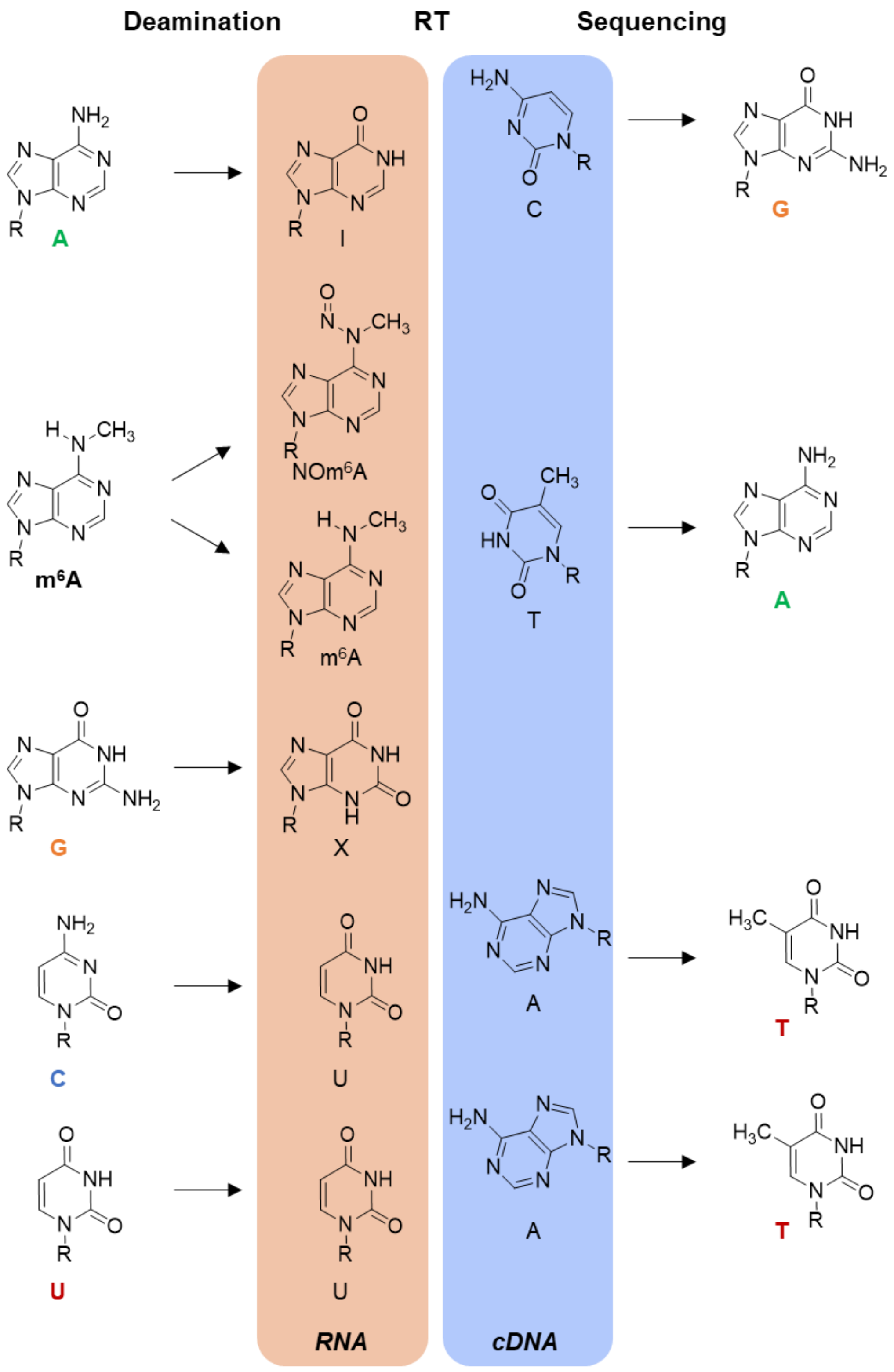

Figure S3. *Base conversion after deamination treatment and following influence on reverse transcription (RT), base-pairing properties and corresponding sequencing output.*

Figure S4

a)

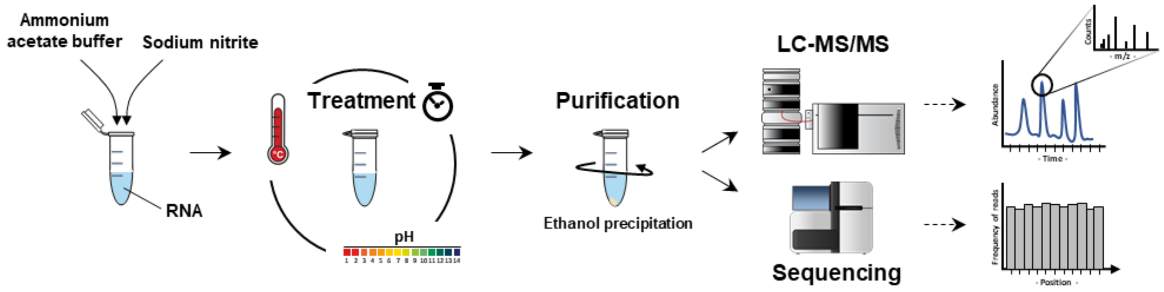

b)

5'-ATAGGGGAATGGGCCGTTCATCTGCTAAAAGGACTGCTTTTGGGGCTTGTAGT-3'

Figure S4. *Deamination treatment and following screening workflow. a) Deamination screening workflow. b) Sequence of synthetic 53mer with m^{6}A at position 33 (red underline).*

Figure S5

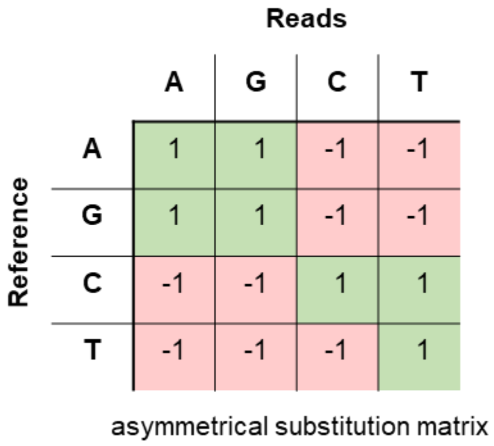

***Figure S5**. Asymmetrical substitution matrix for alignment of partially deaminated reads (green boxes show rewards of +1 for the alignment score, red boxes show penalties of -1 correspondingly).*

Figure S6

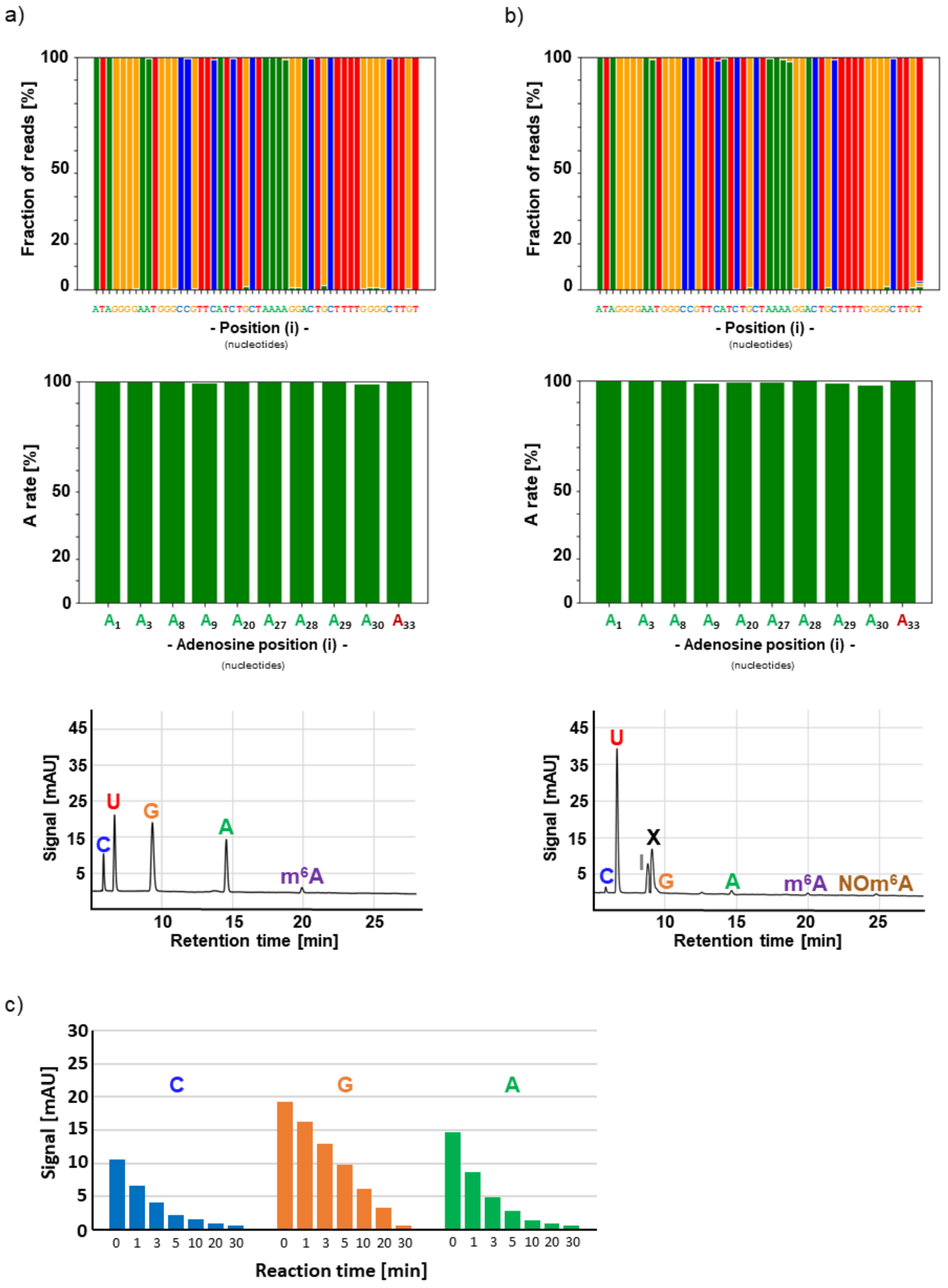

Figure S6. *Strong deamination of the 53mer (70°C, pH 3.5 and 30 min treatment) and the corresponding NOseq and LC-MS data. a) Non-treated reference. Shown are the alignment plot (with fraction of reads, plotted for each position (A in green, G in orange, C in blue and T in red), the adenosine rate plot and the LC-MS chromatogram. b) Strongly deaminated sample. Shown are the alignment plot (with fraction of reads, plotted for each position (A in green, G in orange, C in blue and T in red), the adenosine rate plot and the LC-MS chromatogram. c) LC-MS graph (exemplary for one reaction condition set) showing the course of the deamination reaction of C, G and A under strong deamination conditions (70°C, pH 3.5) at different time points.*

Figure S7

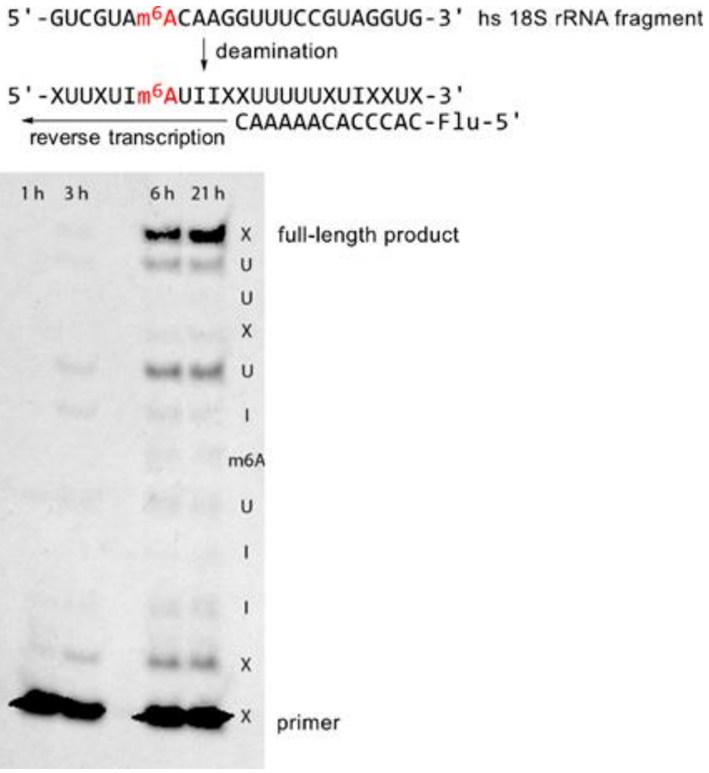

***Figure S7**. Primer extension on a deaminated 24mer fragment of human 18S rRNA containing m^6A_{1832}. The RNA was treated with 1 M diethylene glycol dinitrite in 2 M pyridine, 1 M KSCN, at 37°C for 5 h and isolated by ethanol precipitation. Digestion and analysis by RP-HPLC revealed that all adenosines and cytidines were deaminated to inosine and uridine, respectively, and 50% of guanosines were converted to xanthosines. A 5'-fluorescein-labelled primer complementary to the deaminated RNA was annealed, and primer extension reaction with 0.5 mM dNTPs and 0.1 U/µL M-MuLV RT was incubated at room temperature for up to 21 hours. Aliquots were analyzed on a 20% denaturing PAGE (7 M urea) gel at 35 W. The full-length extension product was produced only after prolonged incubation time and significant abort bands were visible before xanthosines.*

Figure S8

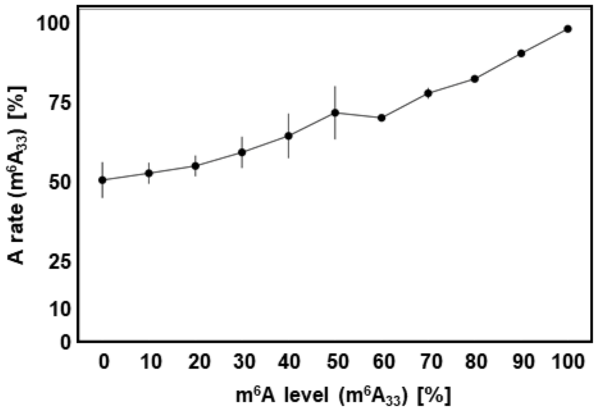

Figure S8*. NOseq data comparison to investigate the quantification of previously detected m^{6}A sites by calibration with deaminated synthetic oligonucleotide mixtures (50°C, pH 4.0 and 20 min), containing ascending m^{6}A contents at position 33 (m^{6}A/A) of the 53mer that was varied from 0 to 100%. The A rate [%] was plotted. Data was averaged from triplicates (error bars show standard deviations; data points for 60, 80 and 90% m^{6}A content derive from one replicate).*

Figure S9

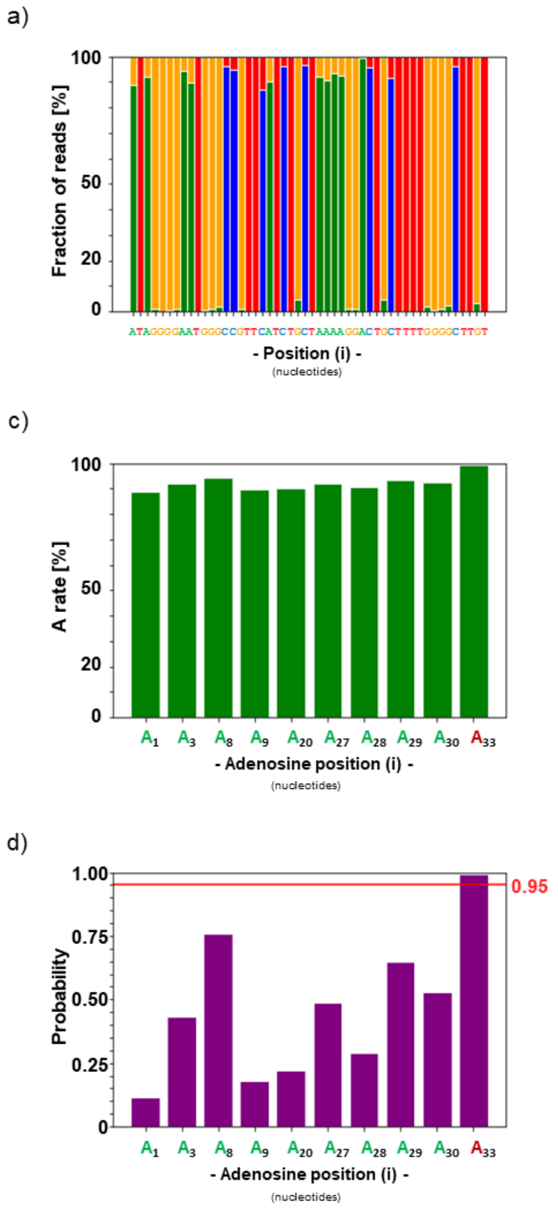

Figure S9. *Plots for deamination condition screening (60°C, pH 5.0 and 10 min treatment) for the 53mer. a) Alignment plot (with fraction of reads in %, plotted for each position (A in green, G in orange, C in blue and T in red). b) Filtered adenosine site alignment plot. c) Adenosine rate and guanosine rate plots (separated adenosine and guanosine fraction of reads in % at A sites). d) Probability plot (red line at 0.95 showing the detection threshold).*

Figure S10

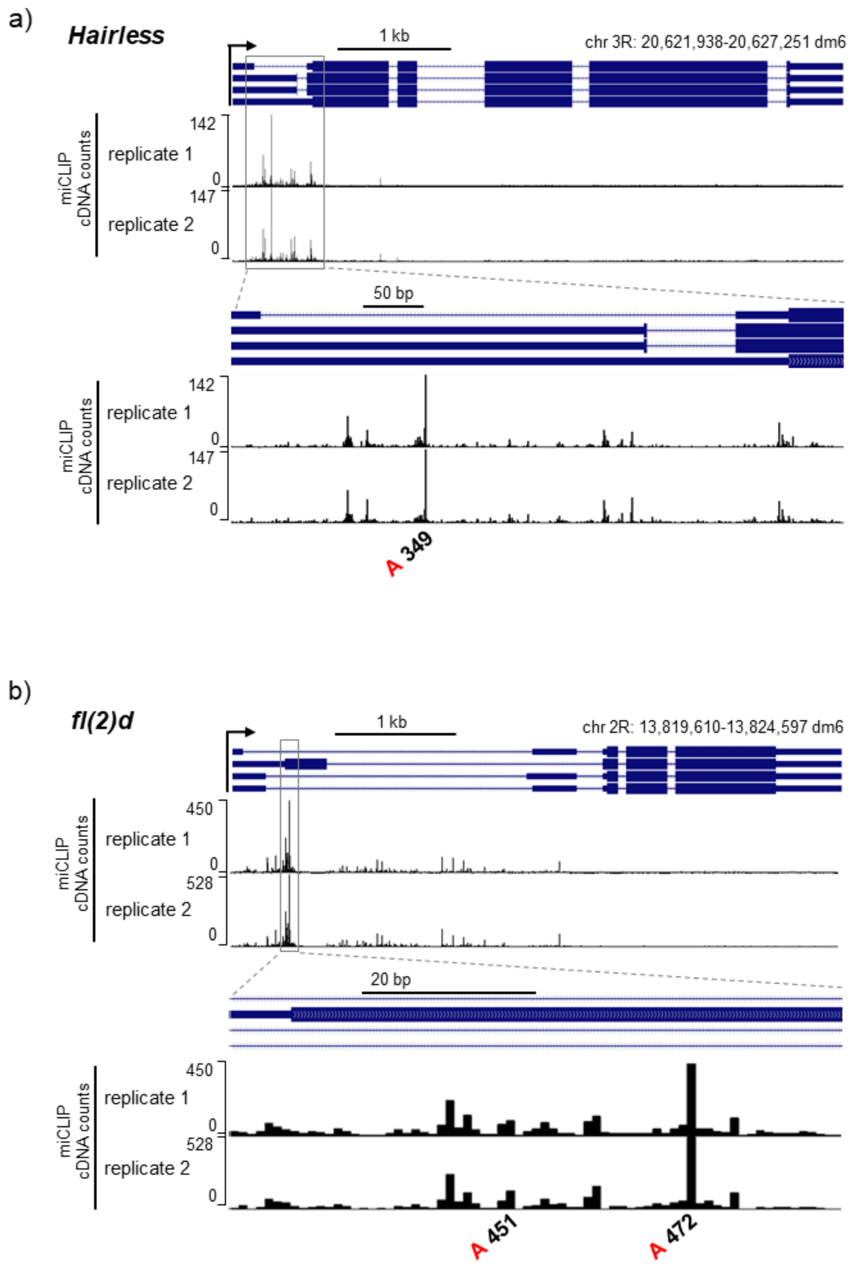

Figure S10. *UCSC Genome Browser screenshots of Hairless and fl(2)d transcript showing miCLIP tracks with cDNA counts of predicted m*^{6}*A positions. a) Gene architecture of Hairless is shown on top, with thin blue boxes representing the 5' and 3' UTRs, thick blue boxes representing the CDS, and thin lines representing introns. Zoom-in view of selected region is shown below with indicated miCLIP cDNA count peak signal of putative m*^{6}*A site (*A_{349}*). b) Gene architecture of fl(2)d is shown on top, with thin blue boxes representing the 5' and 3' UTRs, thick blue boxes representing the CDS, and thin lines representing introns. Zoom-in view of selected region is shown below with indicated miCLIP cDNA count peak signal of two putative m*^{6}*A sites (*A_{451} *and* A_{472}*).*

Figure S11

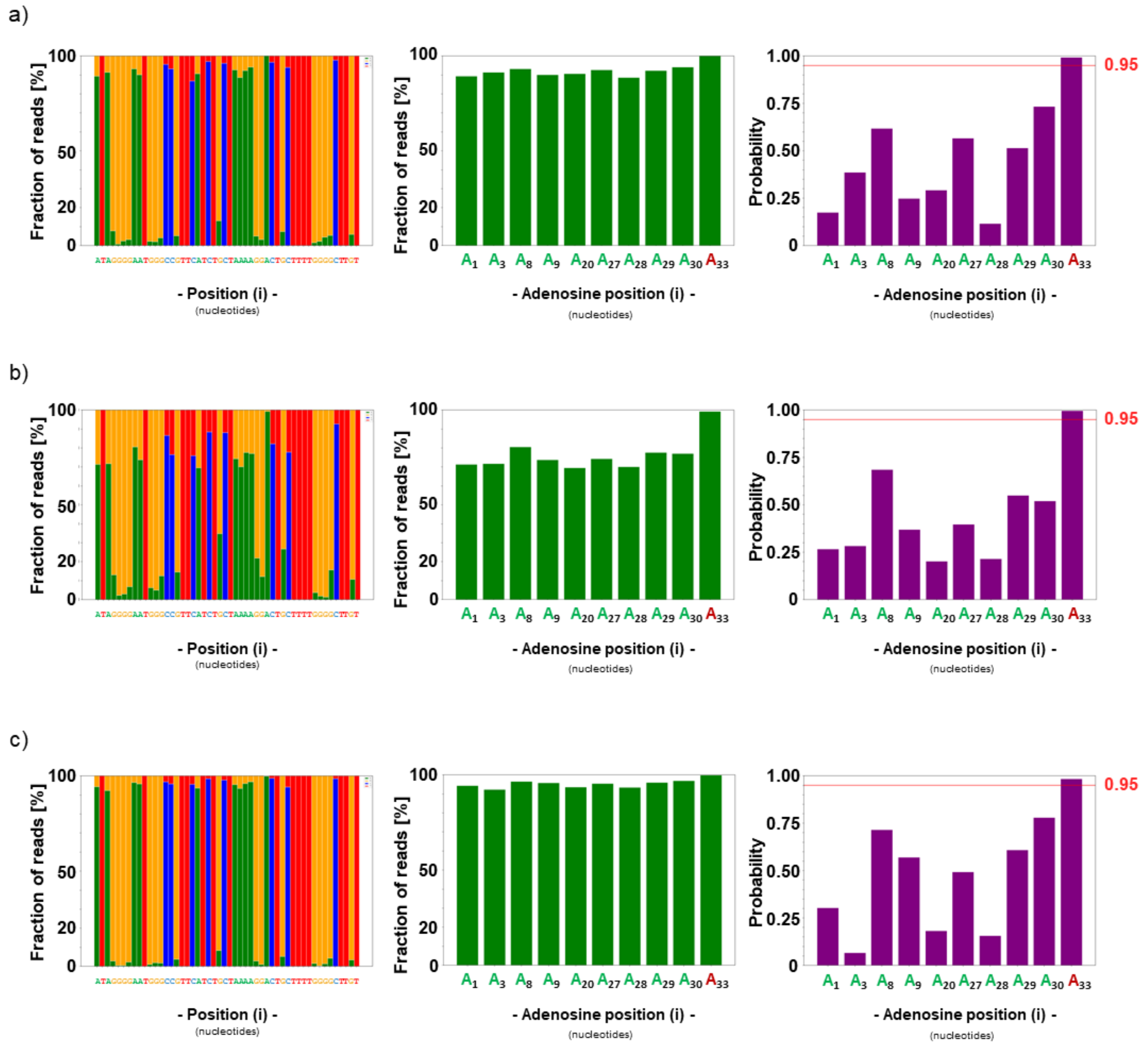

Figure S11. *Plots for deamination condition screening (50°C, pH 4.0) for the 53mer with SuperScript IV (Thermo Fisher Scientific). a) Alignment plot (with fraction of reads in %, plotted for each position (A in green, G in orange, C in blue and T in red), Adenosine rate plot (separated adenosine fraction of reads in % at A sites) and probability plot (red line at 0.95 showing the detection threshold) for 3 min treatment. b) Alignment plot (with fraction of reads in %, plotted for each position (A in green, G in orange, C in blue and T in red), Adenosine rate plot (separated adenosine fraction of reads in % at A sites) and probability plot (red line at 0.95 showing the detection threshold) for 10 min treatment. c) Alignment plot (with fraction of reads in %, plotted for each position (A in green, G in orange, C in blue and T in red), Adenosine rate plot (separated adenosine fraction of reads in % at A sites) and probability plot (red line at 0.95 showing the detection threshold) for 30 min treatment. RT was performed according to the manufacturer's manual.*

Figure S12

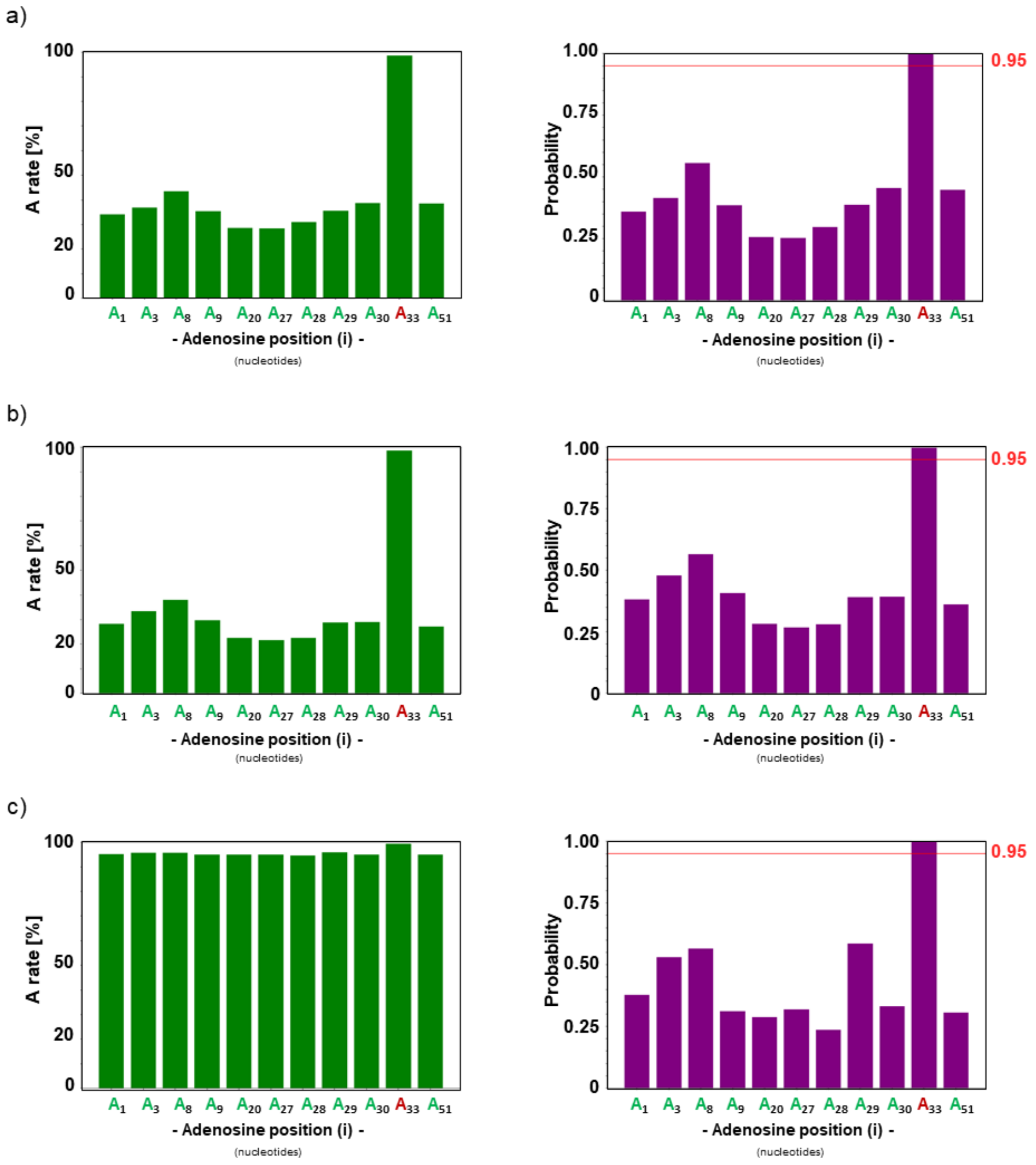

Figure S12. *Deamination of 53mer with diethylene glycol dinitrite. Shown are the adenosine rate (separated adenosine fraction of reads in % at A sites) and probability plots. a) 2 h treatment. b) 5 h treatment. c) 10 h treatment.*

Figure S13

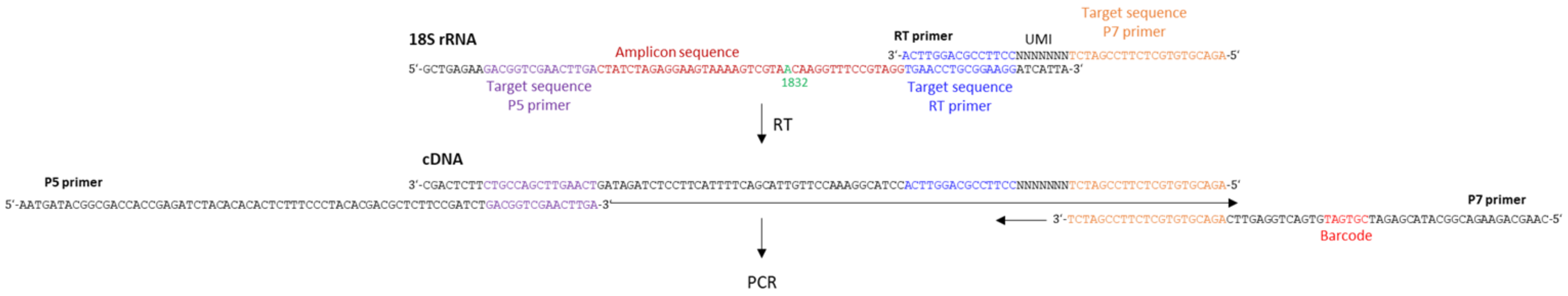

Required primers:

<u>Target specific</u>

RT primer: 5'-AGACGTGTGCTCTTCCGATCTNNNNNNNCCTTCCGCAGGTTCA-3

P5 primer: 5'-AATGATACGGCGACCACCGAGATCTACACACTCTTTCCCTACACGACGCTCTTCCGATCTGACGGTCGAACTTGA-3'

<u>Universal</u> (from NEB Next Multiplex Small RNA Library Prep Set)

P7 primer: 5'-CAAGCAGAAGACGGCATACGAGATCGTGATGTGACTGGAGTTCAGACGTGTGCTCTTCCGATCT-3'

Figure S13. *Primer design for a typical amplicon (red) for amplicon sequencing (exemplary for m^6A_{1832} in human 18S rRNA). Shown are the target-specific sequences (purple and blue) around the m^6A site (green) in the amplicon sequence (red). Shown in blue is the target sequence for the RT primer, containing the unique molecular identifier and the universal target sequence for the P7 primer (orange) from the NEBNext Multiplex Small RNA Library Prep Set. After RT, the target specific P5 primer and the universal P7 primer (including the barcode sequence) are added and the amplicon is amplified via PCR. For other m^6A sites, the target-specific sequences for the RT primer (blue) and the P5 primer (purple) need to be adapted (see primer sequences in Table 2). The amplicon sequences containing the other investigated m^6A candidate sites can be found in Supplement Table S1).*

Figure S14

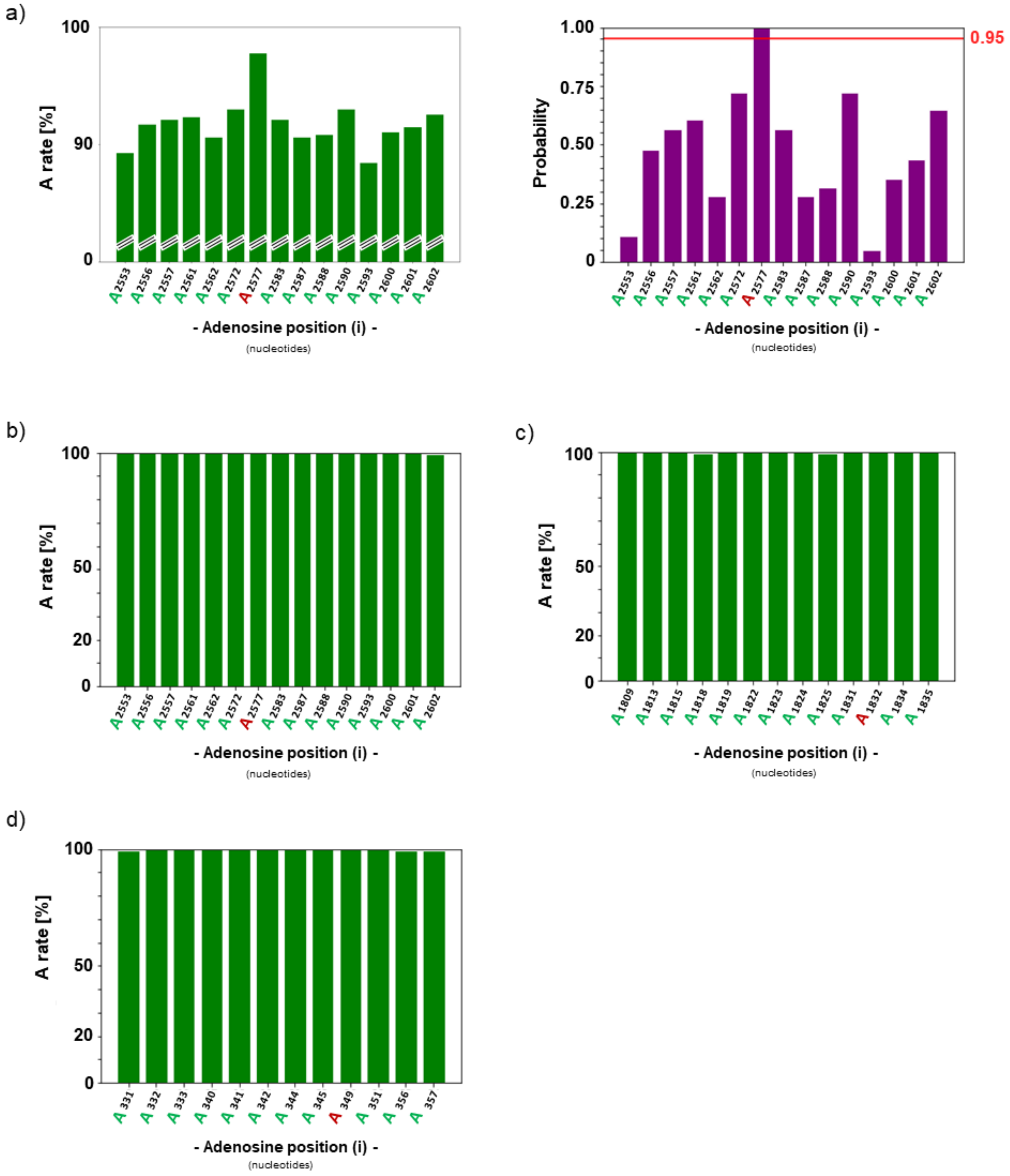

Figure S14. *Additional data for biological samples. a) Adenosine rate (separated adenosine fraction of reads in % at A sites) and probability plot of human lncRNA MALAT1 (HEK293T cells). b) Adenosine rate plot of the non-treated reference of human lncRNA MALAT1 (HeLa cells). c) Adenosine rate plot of the non-treated reference of human 18S rRNA (HEK293T cells). d) Adenosine rate plot of the Drosophila melanogaster H non-treated reference after MeRIP enrichment.*

Figure S15

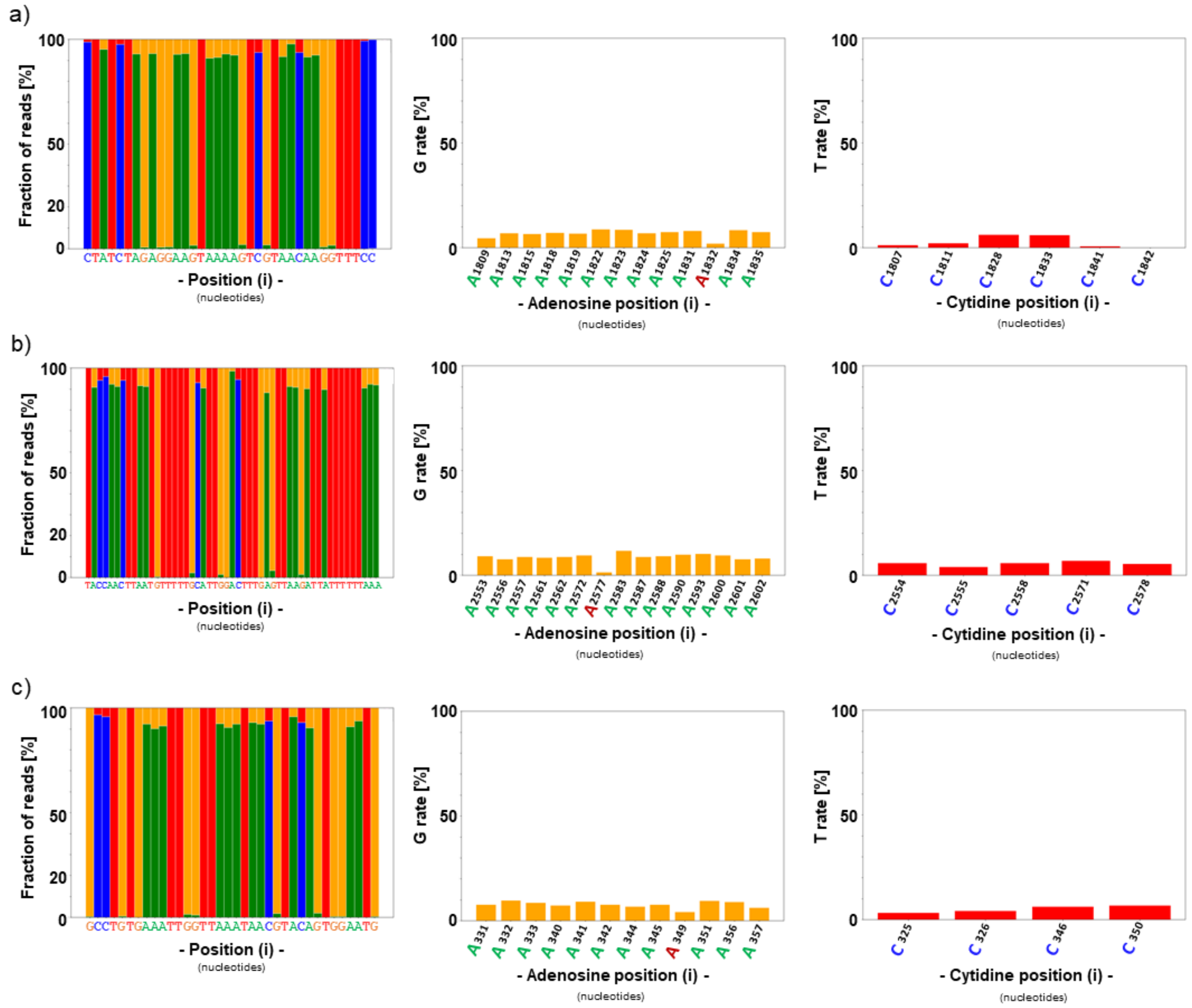

Figure S15. *NOseq data for biological samples. a) 18S rRNA. Alignment (with fraction of reads in %, plotted for each position (A in green, G in orange, C in blue and T in red), Guanosine rate (separated Guanosine fraction of reads in % at A sites) and T rate plot (separated Thymidine fraction of reads in % at C sites). b) MALAT1 lncRNA. Alignment (with fraction of reads in %, plotted for each position (A in green, G in orange, C in blue and T in red), Guanosine rate (separated Guanosine fraction of reads in % at A sites) and T rate plot (separated Thymidine fraction of reads in % at C sites). c) H mRNA. Alignment (with fraction of reads in %, plotted for each position (A in green, G in orange, C in blue and T in red), Guanosine rate (separated Guanosine fraction of reads in % at A sites) and T rate plot (separated Thymidine fraction of reads in % at C sites).*

Figure S16

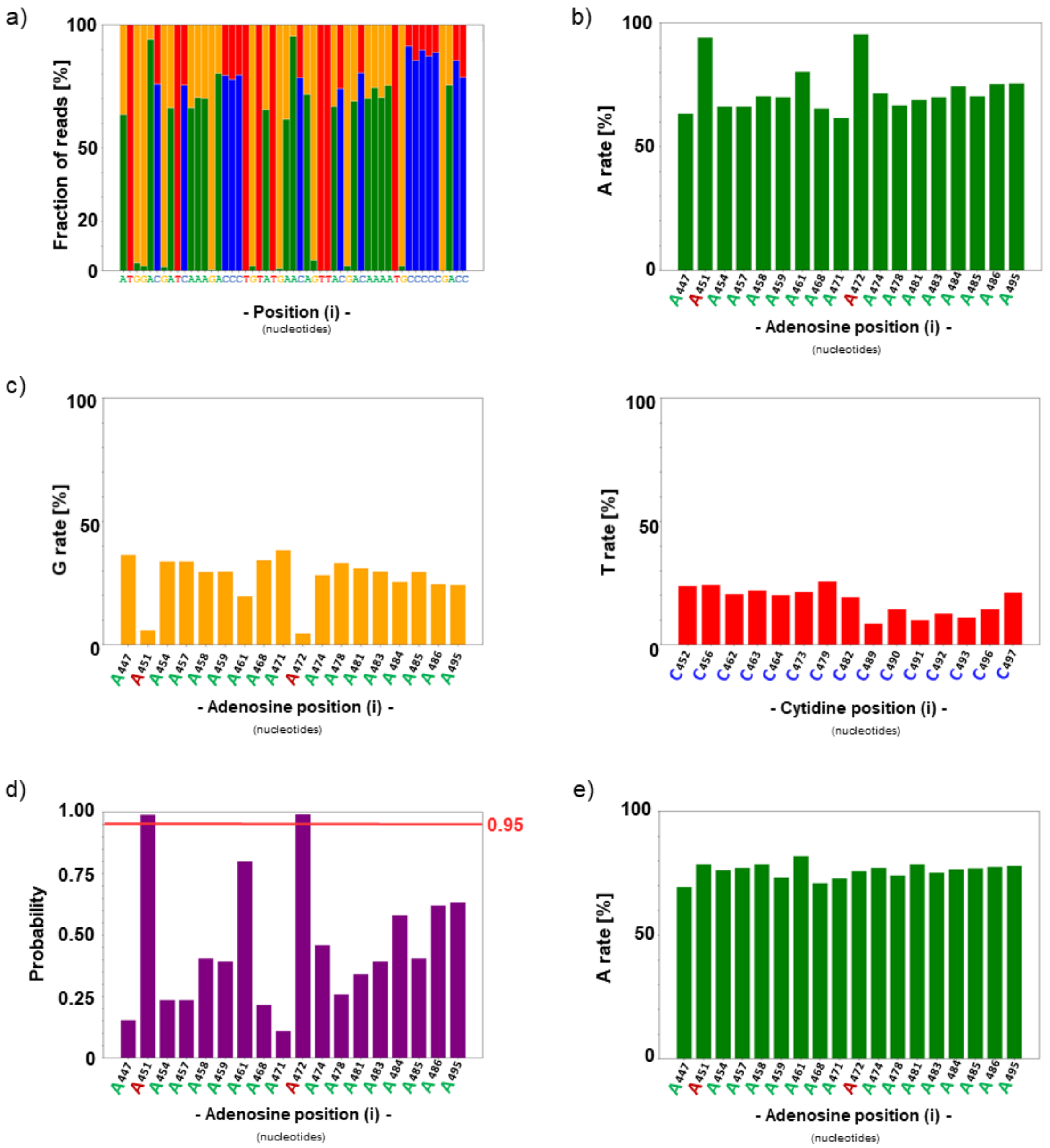

***Figure S16**. NOseq data for Drosophila fl(2)d mRNA (from wildtype flies for a) - d), and IME4- knockout flies for e)). a) Alignment plot (with fraction of reads in %, plotted for each position (A in green, G in orange, C in blue and T in red). b) Adenosine rate (separated Adenosine fraction of reads in % at A sites). c) Guanosine rate (separated Guanosine fraction of reads in % at A sites) and T rate plot (separated Thymidine fraction of reads in % at C sites). d) Probability plot (red line at 0.95 showing the detection threshold). e) Adenosine rate plot (separated Adenosine fraction of reads in % at A sites) of Drosophila fl(2)d mRNA (from IME4-knockout flies).*

Figure S17

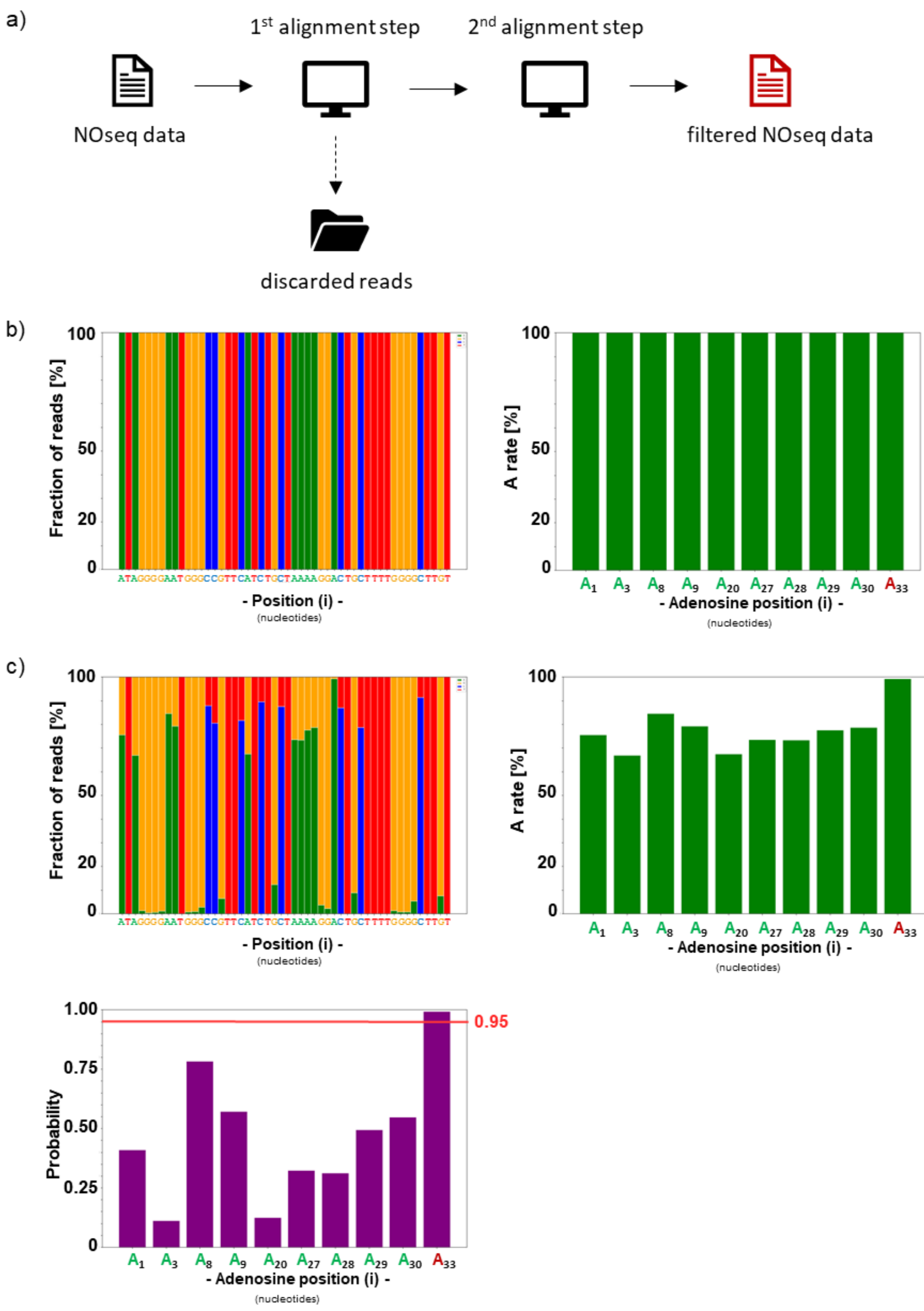

Figure S17*. NOseq alignment test (two-step alignment), exemplary for deamination of the 53mer with a treatment of 50°C, pH 4.0 and 20 min (for comparison to standard alignment - see Figure 2). a) Analytical pipeline. NOseq data is aligned in a 1st alignment step to the reference sequence (allowing no mismatches and no multiple alignment). Perfectly aligned reads are filtered out and discarded from the dataset. The reduced dataset is then aligned in a 2nd alignment step with the NOseq alignment, according to the asymmetric substitution matrix and the custom alignment pipeline (Supplement Figure S5). b) Filtered, discarded reads from 1st alignment step. Alignment (with fraction of reads in %, plotted for each position (A in green, G in orange, C in blue and T in red) and Adenosine rate plot (separated Adenosine fraction of reads in % at A sites). c) Reduced dataset used in 2nd alignment step. Alignment (with fraction of reads in %, plotted for each position (A in green, G in orange, C in blue and T in red), Adenosine rate (separated Adenosine fraction of reads in % at A sites) and Probability plot (red line at 0.95 showing the detection threshold).*
